# Natural Variation in *Arabidopsis* Cvi-0 Accession Reveals an Important Role of MPK12 in Guard Cell CO_2_ Signaling

**DOI:** 10.1371/journal.pbio.2000322

**Published:** 2016-12-06

**Authors:** Liina Jakobson, Lauri Vaahtera, Kadri Tõldsepp, Maris Nuhkat, Cun Wang, Yuh-Shuh Wang, Hanna Hõrak, Ervin Valk, Priit Pechter, Yana Sindarovska, Jing Tang, Chuanlei Xiao, Yang Xu, Ulvi Gerst Talas, Alfonso T. García-Sosa, Saijaliisa Kangasjärvi, Uko Maran, Maido Remm, M. Rob G. Roelfsema, Honghong Hu, Jaakko Kangasjärvi, Mart Loog, Julian I. Schroeder, Hannes Kollist, Mikael Brosché

**Affiliations:** 1 Institute of Technology, University of Tartu, Tartu, Estonia; 2 Division of Plant Biology, Department of Biosciences, Viikki Plant Science Centre, University of Helsinki, Helsinki, Finland; 3 Division of Biological Sciences, Cell and Developmental Biology Section, University of California, San Diego, La Jolla, California, United States of America; 4 College of Life Science and Technology, Huazhong Agricultural University, Wuhan, China; 5 Institute of Molecular and Cell Biology, University of Tartu, Tartu, Estonia; 6 Institute of Chemistry, University of Tartu, Tartu, Estonia; 7 Molecular Plant Biology, Department of Biochemistry, University of Turku, Turku, Finland; 8 Molecular Plant Physiology and Biophysics, Julius-von-Sachs Institute for Biosciences, Biocenter, University of Würzburg, Würzburg, Germany; 9 Distinguished Scientist Fellowship Program, College of Science, King Saud University, Riyadh, Saudi Arabia; Duke University, United States of America

## Abstract

Plant gas exchange is regulated by guard cells that form stomatal pores. Stomatal adjustments are crucial for plant survival; they regulate uptake of CO_2_ for photosynthesis, loss of water, and entrance of air pollutants such as ozone. We mapped ozone hypersensitivity, more open stomata, and stomatal CO_2_-insensitivity phenotypes of the *Arabidopsis thaliana* accession Cvi-0 to a single amino acid substitution in MITOGEN-ACTIVATED PROTEIN (MAP) KINASE 12 (MPK12). In parallel, we showed that stomatal CO_2_-insensitivity phenotypes of a mutant *cis* (CO_2_-insensitive) were caused by a deletion of *MPK12*. Lack of MPK12 impaired bicarbonate-induced activation of S-type anion channels. We demonstrated that MPK12 interacted with the protein kinase HIGH LEAF TEMPERATURE 1 (HT1)—a central node in guard cell CO_2_ signaling—and that MPK12 functions as an inhibitor of HT1. These data provide a new function for plant MPKs as protein kinase inhibitors and suggest a mechanism through which guard cell CO_2_ signaling controls plant water management.

## Author Summary

Human activities have increased the concentrations of CO_2_ and harmful air pollutants such as ozone in the troposphere. These changes can have detrimental consequences for agricultural productivity. Guard cells, which form stomatal pores on leaves, regulate plant gas exchange. To maintain photosynthesis, stomata open to allow CO_2_ uptake, but at the same time, open stomata lead to loss of water and allow the entrance of ozone. Elevated atmospheric CO_2_ levels reduce stomatal apertures, which can improve plant water balance but also increases leaf temperature. Using genetic approaches—in which we exploit natural variation and mutant analysis of thale cress (*Arabidopsis thaliana*)—we find that MITOGEN-ACTIVATED PROTEIN KINASE 12 (MPK12) and its inhibitory interaction with another kinase, HIGH LEAF TEMPERATURE 1 (HT1) (involved in guard cell CO_2_ signaling), play a key role in this regulatory process. We have therefore identified a mechanism in which guard cell CO_2_ signaling regulates how efficiently plants use water and cope with the air pollutant ozone.

## Introduction

Human activities have increased the concentrations of CO_2_ and harmful air pollutants such as ozone in the troposphere. During the last 200 y, the CO_2_ concentration has increased from 280 to 400 ppm, and it is predicted to double relative to the preindustrial level by 2050 [1]. Elevated CO_2_ is likely to have complex effects on plant productivity, since CO_2_ is not only a driver of climate change but also the main substrate for photosynthesis. Altered atmospheric chemistry is not limited to CO_2;_ the concentration of tropospheric ozone has more than doubled within the past 100 y [2]. Ozone is a notorious air pollutant causing severe damage to crops; present day global yield reductions caused by ozone range from 8.5%–14% for soybean, 3.9%–15% for wheat, and 2.2%–5.5% for maize [3]. Both CO_2_ and ozone enter the plant through stomata—small pores on the surfaces of plants that are formed by pairs of guard cells. Guard cells also regulate plant water balance since plants with more open stomata allow faster water evaporation. Water availability is the most limiting factor for agricultural production, and insufficient water supply can cause large reductions in crop yields [4]. Thus, plants are constantly facing a dilemma; assimilation of CO_2_ requires stomatal opening but also opens the gates for entrance of harmful air pollutants and leads to excessive water loss. A consequence of increased atmospheric CO_2_ concentration can be higher biomass production [5], but at the same time, plants adjust to elevated CO_2_ by partial closure of stomata [5,6] and expressing an altered developmental program that leads to reduced stomatal number [7]. CO_2_-induced stomatal closure reduces water loss; hence, it can directly modify plant water use efficiency (WUE)—carbon assimilated through photosynthesis versus water lost through stomata.

Natural variation among *Arabidopsis thaliana* accessions provides a rich genetic resource for addressing plant function and adaptation to diverse environmental conditions. The *Arabidopsis* accession Cvi-0 from the Cape Verde Islands has impaired CO_2_ responses, more open stomata than Col-0, and is extremely sensitive to ozone treatment [8,9]. A single amino acid change in Cvi-0 MITOGEN-ACTIVATED PROTEIN KINASE 12 (MPK12) was recently shown to affect water use efficiency as well as stomatal size and to impair abscisic acid (ABA)-induced inhibition of stomatal opening [10]. MPK12 also regulates auxin signaling in roots [11]. However, the involvement of MPK12 in the CO_2_ signaling pathway in guard cells has not been addressed thus far.

Among the important components of *A*. *thaliana* guard cell CO_2_ signaling are carbonic anhydrases (βCA1 and βCA4) that catalyze the conversion of CO_2_ to bicarbonate and the protein kinase HIGH LEAF TEMPERATURE 1 (HT1) that has been suggested to function as a negative regulator of CO_2_-induced stomatal movements [12,13]. Ultimately, for stomata to close, a signal from the bicarbonate has to activate protein kinases such as OPEN STOMATA 1 (OST1) that in turn activate plasma membrane anion channels, including SLOW ANION CHANNEL 1 (SLAC1), followed by extrusion of ions and water that causes stomatal closure [14–17]. Isolation of a dominant *HT1* allele, *ht1-8D*, revealed that HT1 may directly inhibit OST1- and GUARD CELL HYDROGEN PEROXIDE-RESISTANT 1 (GHR1)-induced activation of SLAC1 [18]. Bicarbonate-induced activation of SLAC1 has been reconstituted in *Xenopus laevis* oocytes [19,20]. The pathway was shown to consist of RESISTANT TO HIGH CARBON DIOXIDE 1 (RHC1), HT1, OST1, and SLAC1 [19], while more recently the importance of CARBONIC ANHYDRASE 4 (βCA4), aquaporin PIP2;1, OST1, and SLAC1 was demonstrated [20]. Although guard cells are perhaps the best characterized single cell signaling system in the plant kingdom, there are still large gaps in our understanding of how CO_2_ signaling in guard cells is regulated and by which mechanism CO_2_ might regulate plant water management and WUE [5,21,22].

Here, we present the results of quantitative trait loci (QTL) mapping and sequencing of near-isogenic lines (NILs) of Cvi-0 ozone sensitivity. In a parallel approach, we mapped more open stomata and CO_2_-insensitivity phenotypes of a mutant *cis* (CO_2_ insensitive). A single amino acid change (G53R) in MPK12 and complete deletion of *MPK12* are the causes of more open stomata and altered CO_2_ responses of Cvi-0 and *cis*, respectively. Based on kinase activity assays, we conclude that MPK12 acts as an inhibitor of the HT1 kinase, which represents a crucial step in the regulation of plant stomatal CO_2_ responses.

## Results

### Mapping of Cvi-0 Ozone Sensitivity Phenotypes

**Fig 1:**
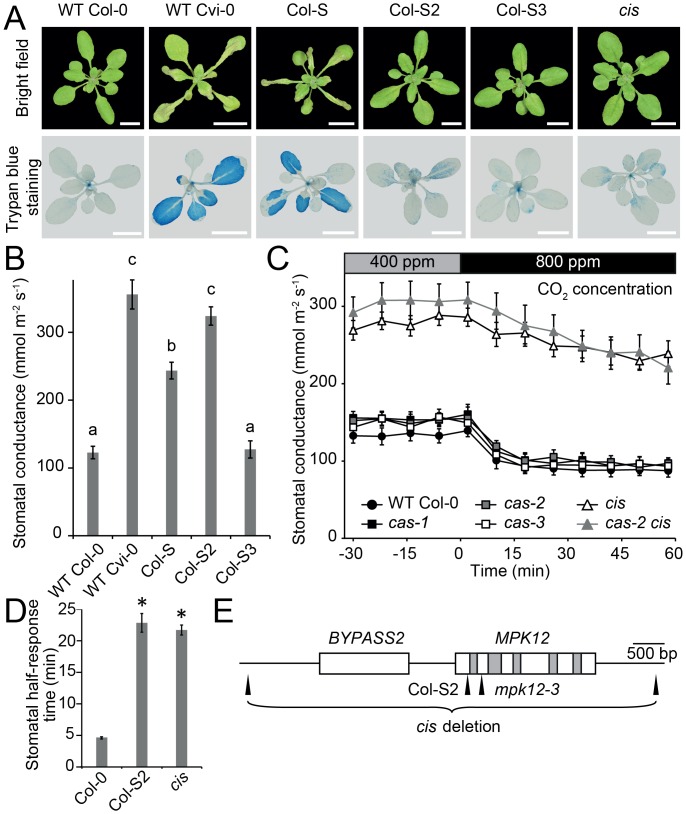
Mapping Cvi-0 ozone sensitivity and Cvi-0 and *cis* stomatal phenotypes. (A) Tissue damage after 6 h of O_3_ exposure (350 ppb). Visual damage of plant rosettes (upper images) and cell death visualized with trypan blue staining (lower images). Scale bars 1 cm. (B) Stomatal conductance of Col-0, Cvi-0, and NILs (mean ± standard error of the mean [SEM], *n* = 7–12). (C) Elevated CO_2_ (800 ppm) induced stomatal closure in intact whole plants (*n* = 9–10, except *cas-2* [*n* = 3]). Experiment was repeated at least three times with similar results. (D) Stomatal half-response times to elevated CO_2_ (800 ppm). Error bars indicate ± SEM (*n* = 13). Pooled data from two experimental series are shown. (E) Gene model of *MPK12* (At2g46070) and *BYPASS2* (At2g46080). The deletion mutant *cis* (renamed as *mpk12-4*) has a 4,772 bp deletion (end and start indicated). Col-S2 has a G to C missense mutation at position 157 of *MPK12*, which leads to G53R substitution in MPK12. The *mpk12-3* mutant has a Syngenta Arabidopsis Insertion Library (SAIL) transfer DNA (T-DNA) insertion in the second exon of *MPK12*. White boxes refer to exons, grey boxes to introns, and black lines to intergenic regions. Small letters (B) and asterisks (D) denote statistically significant differences according to one-way ANOVA with Tukey honest significant difference (HSD) for unequal sample size (Spjotvoll & Stoline test) or Tukey HSD post hoc test, respectively. The raw data for panels B–D can be found in S1 Data.

Our initial QTL mapping of ozone sensitivity in Cvi-0 placed the two major contributing loci on the lower ends of chromosomes 2 and 3 [8]. To identify the causative loci related to the extreme ozone sensitivity and more open stomata of Cvi-0, we created a NIL termed Col-S (for Col-0 ozone sensitive) through eight generations of backcrossing of Cvi-0 with Col-0 (Fig 1A, S1A Fig, and S1 Video). In parallel, ozone tolerance from Col-0 was introgressed to Cvi-0 by six generations of backcrossing, which generated the ozone-tolerant Cvi-T (S1 Video). Using these accessions, NILs, and recombinant inbred lines (RILs), we mapped the causative ozone QTLs to a region of 90 kb on chromosome 2 and 17.70–18.18 Mbp on chromosome 3 (S1B Fig). We have previously shown that the QTL on chromosome 2 also controls plant water loss and stomatal function [8]. We isolated both QTLs by backcrossing Col-S with Col-0 and obtained the NILs Col-S2 and Col-S3. Both of these were less sensitive to ozone than Col-S (S1A Fig), indicating that these QTLs act additively to regulate ozone sensitivity. Col-S2 (but not Col-S3) showed much higher daytime stomatal conductance than Col-0 (Fig 1B). The mapping resolution on chromosome 3 was not sufficient to identify the causative gene. Hence, we focused on Col-S2 and its role in stomatal function. Within the 90-kb mapping region on chromosome 2, one gene, At2g46070, encoding a MAP kinase MPK12, shows strong preferential guard cell expression [23]. A single point mutation was found in Cvi-0 *MPK12*, leading to a glycine to arginine substitution at position 53 of the protein.

### Stomata-Related Phenotypes of Cvi-0 and *cis* are Caused by Mutations in *MPK12*

CAS (calcium-sensing receptor) is a chloroplast-localized protein important for proper stomatal responses to external Ca^2+^ [24,25]. While testing stomatal phenotypes in *cas* mutants, we observed phenotypic discrepancy between different alleles of *cas*. Whereas the *cas-2* (GABI-665G12) line had more open stomata and impaired CO_2_ responses, this was neither observed in *cas-1* nor in *cas-3* (Fig 1C and S1C Fig). Further experiments showed that the T-DNA insert in the *CAS* gene was not linked to the CO_2_-insensitive phenotype of *cas-2*. In a backcross with Col-0, the T-DNA insert in *cas-2* was removed, thereby generating the mutant *cis* (*C**O*_*2*_ *i**n**s**ensitive*). Both *cis* and Col-S2 had impaired responses to high CO_2_ (800 ppm), leading to longer half-response times, but a residual CO_2_ response could still be observed (Fig 1D and S1E Fig).

In order to identify the causative mutation in *cis*, mapping and whole genome sequencing of *cis* × C24 population was performed, which revealed a complete deletion of the *MPK12* gene and its neighbor *BYPASS2* in *cis* (Fig 1E and S1D Fig). Thus, *cis* was renamed *mpk12-4*. A second mutant (*gdsl3-1*) from the GABI-Kat collection (GABI-492D11) contained an identical deletion of *BYPASS2* and *MPK12* (S2 Fig). We also identified a line with a T-DNA insert in exon 2 of *MPK12* from the SAIL collection (Fig 1E), which was recently named *mpk12-3* [26]. No full-length transcript was found in *mpk12-3* (S3 Fig). SALK T-DNA insertion lines of *MPK12* were previously described as lethal [11,23]; similarly, we were unable to retrieve homozygous plants of the same alleles, possibly indicating the presence of an additional T-DNA in an essential gene. The new *mpk12* deletion, SAIL T-DNA insertion, and Col-S2 point mutation alleles allowed a detailed characterization of the role of MPK12 in stomatal regulation.

**Fig 2:**
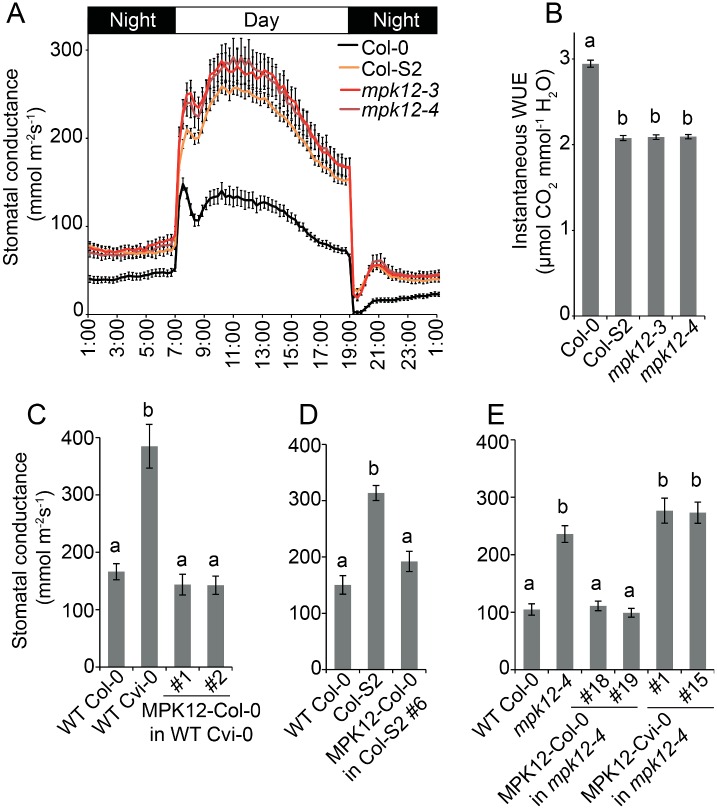
Stomatal conductance of the NIL Col-S2, *mpk12* mutants, and complementation lines. (A) Diurnal pattern of stomatal conductance with 12 h/12 h light–dark periods (*n* = 13–16). (B) Instantaneous water use efficiency (WUE) measured as an average of daytime light period from 09:00 to 17:00 (*n* = 13–16). (C) Stomatal conductance of Cvi-0 transformed with Col-0 *MPK12* driven by its native promoter in T2 generation (*n* = 9). (D) Stomatal conductance of Col-S2 complementation line in T2 generation transformed with Col-0 *MPK12*, driven by its native promoter (*n* = 5–8). (E) Stomatal conductance of T3 transformants in the *mpk12-4* background transformed with either the Col-0 or Cvi-0 version of *MPK12*, driven by its respective native promoter (*n* = 5–6). All graphs present mean ± SEM. Small letters denote statistically significant differences according to one-way ANOVA with Tukey HSD post hoc test for either unequal (B, D, E) or equal sample size (C). The raw data for panels A–E can be found in S1 Data.

Stomatal conductance was higher throughout the day in all three lines (Col-S2, *mpk12-3*, and *mpk12-4*) (Fig 2A), suggesting that the amino acid substitution in Cvi-0 MPK12 leads to loss of function. Furthermore, Col-0 transformed with *MPK12* from Cvi-0 showed stomatal conductance similar to Col-0, which excludes the option that the G53R substitution in MPK12 would lead to gain of function (S1F Fig). Moreover, the wild-type (Col-0) stomatal phenotype was observed in heterozygous F1 plants from a cross of Col-S2 and Col-*gl1*, in which the *gl1* mutation that gives a trichome-less phenotype was used as a noninvasive method for selecting successfully crossed plants in the first generation (S1G Fig). Increased stomatal conductance may result from an increased number of stomata, larger stomata, or more open stomata. However, the stomatal index, length, and density did not differ between the lines, indicating that MPK12 regulates a function related to the stomatal aperture (S4 Fig). Because of the higher degree of stomatal opening, the instantaneous WUE was lower in *mpk12-3*, *mpk12-4*, and Col-S2 (Fig 2B). Altered WUE was previously also seen in *mpk12-1* and a NIL with Cvi-0 MPK12 in L*er* [10].

Cvi-0 and Col-S2 were complemented by expression of MPK12 from Col-0 (Fig 2C and 2D). Similarly, *mpk12-4* was complemented by expression of Col-0 MPK12 but not by Cvi-0 MPK12 (Fig 2E). We conclude that MPK12 is a crucial regulator of stomatal conductance, and a single amino acid substitution (G53R) in Cvi-0 leads to loss of function of MPK12.

### MPK12 Functions in Guard Cell CO_2_ Signaling

**Fig 3:**
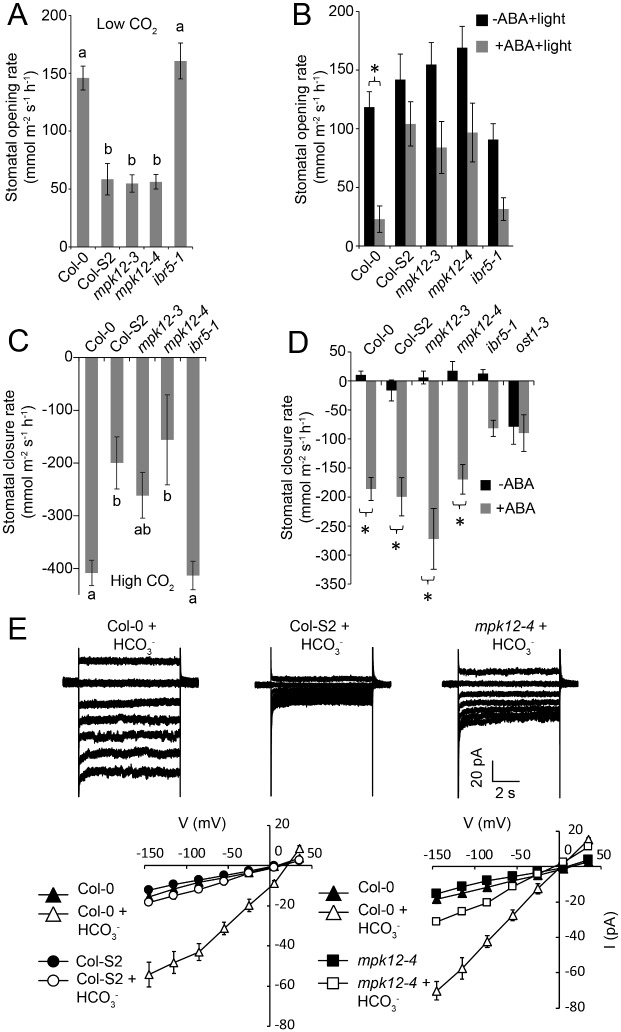
Responsiveness of the NIL Col-S2 and *mpk12* mutants to stomatal opening and closing stimuli. (A) Stomatal opening induced by 100 ppm CO_2_ in whole plants (58 min after induction; *n* = 12–13). (B) Light-induced stomatal opening inhibited by 2.5 μM ABA in whole plants (24 min after induction; *n* = 16–18). (C) Stomatal closure induced by 800 ppm CO_2_ in whole plants (10 min after induction; *n* = 12–13). (D) Stomatal closure induced by spraying whole plants with 5 μM ABA solution (24 min after induction; *n* = 12–14). (E) MPK12 is required for the bicarbonate (HCO_3_^-^)-induced slow type anion channel activation in guard cell protoplasts. Upper panels show typical whole guard cell protoplast recordings with 11.5 mM free HCO_3_^-^ added to the pipette solution, and lower panels show average steady-state current-voltage relationships for wild-type (Col-0), NIL Col-S2, and *mpk12-4* after treatment with mock or 11.5 mM HCO_3_^-^ (*n* = 4–8 per line and treatment). Small letters (A, C) and asterisks (B, D) indicate statistically significant differences according to one-way ANOVA and two-way ANOVA with Tukey HSD for unequal sample size post hoc tests (*p* < 0.05), respectively. Error bars mark ± SEM. The raw data for panels A–E can be found in S1 Data.

Reduction of CO_2_ levels inside the leaf [27] is a signal that indicates a shortage of substrate for photosynthesis and triggers stomatal opening. The rate of stomatal opening in response to low CO_2_ was severely impaired in *mpk12* and Col-S2 (Fig 3A and S5A Fig). Another signal for stomatal opening is light; this response was intact in plants with impaired or absence of MPK12 (S5B and S5C Fig). The hormone ABA has dual roles in stomatal regulation; it induces stomatal closure but also inhibits light-induced stomatal opening. The latter response was impaired in *mpk12* mutants and Col-S2 (Fig 3B and S5C Fig). Stomata close in response to several signals, including darkness, reduced air humidity, ozone pulse, elevated CO_2_, and ABA. Of these, only the response to elevated CO_2_ was impaired in *mpk12* and Col-S2 (Fig 3C and 3D, S5D–S5H and S6 Figs). CO_2_ signaling is impaired in the carbonic anhydrase double mutant *ca1 ca4* [13], and the product of carbonic anhydrase, bicarbonate, activates S-type anion currents [15]. In Col-S2 and *mpk12-4*, bicarbonate-induced S-type anion currents were strongly impaired (Fig 3E). Collectively, these data indicated that MPK12 has an important role in the regulation of CO_2_-induced stomatal movements in *Arabidopsis*.

### MPK12 Interacts with the Protein Kinase HT1

**Fig 4:**
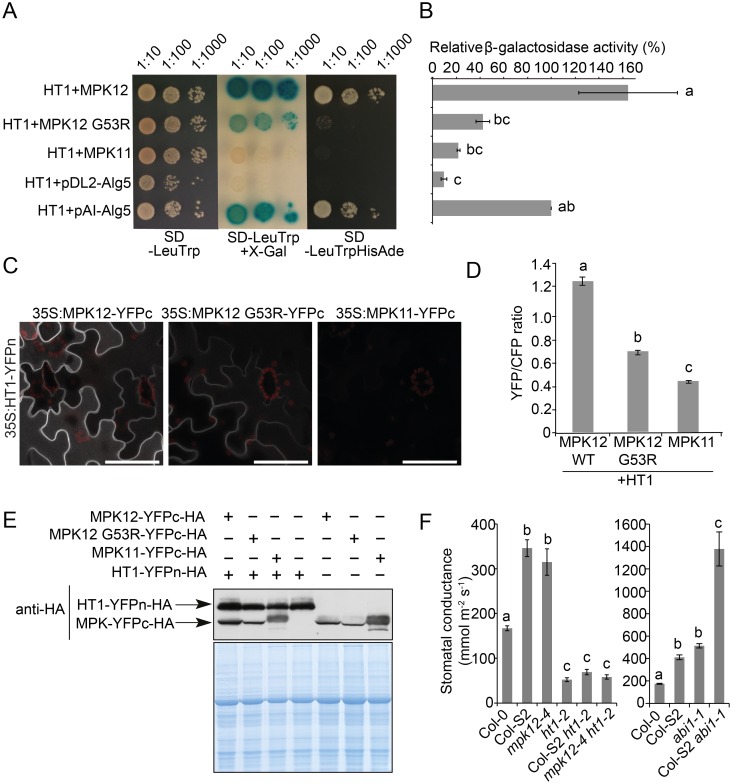
MPK12 interacts with HT1. (A) Split-ubiquitin yeast two-hybrid assay on the SD-LeuTrp plate (left and middle panels) indicates the presence of both bait and prey plasmids; X-gal overlay assay (middle) and growth assay on the SD-LeuTrpHisAde plate (right) show HT1 interaction with MPK12 that is similar to the positive control (pAI-Alg5). Only weak or no interaction was detected with MPK12 G53R and MPK11, similar to the negative control (pDL2-Alg5). (B) Quantitative β-galactosidase assay from pools of ten colonies each. Activities are shown as the percentage of the positive control (± SEM; *n* = 3). (C) High-magnification (63x objective) BiFC images from a single infiltrated *N*. *benthamiana* leaf with identical confocal microscopy acquisition settings. Scale bar = 50 μm. (D) Ratiometric BiFC shows weaker interaction of MPK12 G53R than MPK12 with HT1, while MPK11 exhibits a weak interaction with HT1. The plasma membrane–localized SLAC1-CFP was used as an internal control. Eighteen images (from three leaves) of each construct set were analyzed. (E) Western blot together with Coomassie staining of proteins extracted from BiFC samples used for confocal imaging and controls with single construct shows expression of all fusion proteins. (F) Steady-state stomatal conductance of Col-S2 *ht1-2*, *mpk12-4 ht1-2*, and Col-S2 *abi1-1* (*ABA insensitive 1–1*) double mutants (mean ± SEM, *n* = 11–13). Experiments were repeated at least three times. Letters in B, D, and F denote statistically significant differences with one-way ANOVA and Tukey HSD post hoc test for equal B, D, or unequal F sample size. The raw data for panels B, D, and F can be found in S1 Data.

Only a few regulators of stomatal CO_2_ signaling in *Arabidopsis* have been identified. These include the protein kinases HT1 and OST1 [12,15,16]. To find the interaction partners of MPK12, we conducted pairwise split-ubiquitin yeast two-hybrid (Y2H) assays against several kinases and phosphatases involved in stomatal signaling (Fig 4A and 4B and S7A and S7B Fig). A strong interaction was observed between MPK12 and HT1 in yeast. The MPK12–HT1 interaction was also confirmed in *Nicotiana benthamiana* with bimolecular fluorescence complementation (BiFC) (Fig 4C–4E) and split luciferase complementation assays (S7C Fig). Strong interaction between MPK12 and HT1 was observed in the cell periphery (Fig 4C). Recently, HT1 was shown to be a plasma membrane–associated protein [28]. In contrast, Col-0 and Cvi-0 MPK12-YFP were located inside the cell (S8A–S8D Fig). Hence, it is likely that the interaction with HT1 brings MPK12 to the plasma membrane. HT1 interacted with both the Col-0 and Cvi-0 versions of MPK12, but the interaction with Cvi-0 MPK12 (G53R) was weaker both in quantitative BiFC and Y2H assays (Fig 4B and 4D). MPK11, an MPK from the same group as MPK12 [29], did not interact with HT1 (Fig 4C). INDOLE-3-BUTYRIC ACID RESPONSE 5 (IBR5) is a MPK phosphatase that regulates auxin signaling in roots and has been shown to interact with and regulate the activity of MPK12 [11]. We confirmed the interaction between MPK12 and IBR5 (S7A Fig). However, the *ibr5-1* mutant exhibited wild-type stomatal phenotypes in response to CO_2_ changes (Fig 3A and 3C, and S5A and S5E Fig), suggesting that IBR5 is not required in stomatal CO_2_ signaling.

### MPK12 Inhibits HT1 Activity

The function of MPK12 in ABA and CO_2_ signaling was further explored through genetic analysis. A strong loss-of-function allele, *ht1-2*, that has low stomatal conductance [12] was used to evaluate the relationship between *mpk12* and *ht1-2*. The Col-S2 *ht1-2* and *mpk12-4 ht1-2* double mutants had a more closed stomata phenotype similar to *ht1-2* (Fig 4F), suggesting that *HT1* is epistatic to *MPK12*. The strong impairment of stomatal function in *abi1*-*1* (*ABA insensitive1-1*) was additive to Col-S2 in the double mutant Col-S2 *abi1-1* (Fig 4F). Hence, signaling through MPK12 seems to act—at least to some extent—independently of the core ABA signaling pathway.

**Fig 5:**
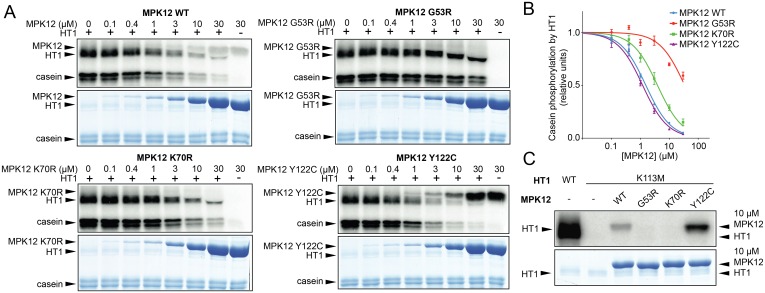
Regulation of HT1 by MPK12. (A) Inhibition of HT1 kinase activity in vitro by different versions of MPK12 (MPK12 G53R—Cvi-0 version of MPK12; MPK12 K70R—inactive kinase; MPK12 Y122C—hyperactive kinase). Upper panel: autoradiography of the SDS PAGE gel; lower panel: Coomassie-stained SDS PAGE. Reaction mixture was incubated for 30 min. (B) Casein phosphorylation by HT1 with different MPK12 concentrations (mean ± SEM; *n* = 3). The raw data can be found in S1 Data. (C) Kinase-dead HT1 K113M was not in vitro phosphorylated by different versions of MPK12, and only MPK12 and MPK12 (Y122C) display clear autophosphorylation activities.

Taken together, the MPK12-HT1 interaction (Fig 4A–4E) and the epistasis between *ht1-2* and *mpk12-4* (Fig 4F) suggest that MPK12 functions upstream of HT1 and could regulate the activity of HT1. To test this directly, we performed in vitro kinase assays with casein as the substrate for HT1 (Fig 5A). HT1 displayed strong autophosphorylation and phosphorylated casein efficiently. Addition of the Col-0 version of MPK12 and a hyperactive version (MPK12 Y122C) efficiently inhibited HT1 activity (Fig 5A and quantified in Fig 5B). A point-mutated version (MPK12 K70R) designed to remove the kinase activity of MPK12 also inhibited the autophosphorylation activity of HT1 and phosphorylation of casein by HT1, although it was less efficient than the wild-type (Fig 5B). Importantly, the Cvi-0 version of MPK12 (G53R) displayed strongly suppressed inhibition of HT1 activity (Fig 5A and 5B). MPK12 did not phosphorylate the kinase-dead version of HT1 (K113M) (Fig 5C). The kinase-dead version of HT1 (K113M) was used as a substrate, since the strong autophosphorylation activity of HT1 would otherwise have obscured the result. Wild-type MPK12 and hyperactive MPK12 (Y122C) displayed autophosphorylation, whereas MPK12 (G53R) as well as MPK12 (K70R) had lost their autophosphorylation activity, indicating that the G53R substitution in Cvi-0 MPK12 disrupts the kinase activity of the protein (Fig 5C). The inhibition of HT1 by MPK12 was specific, as MPK11, which belongs to the same group as MPK12, was not able to affect HT1 kinase activity (S9 Fig). We conclude that the stomatal phenotypes of *mpk12* mutants and Cvi-0 can be explained by a lack of inhibition of HT1 activity by MPK12, which leads to more open stomata and impaired CO_2_ responses (Figs 2A, 3 and 5A and 5C).

### Both MPK12 and MPK4 Regulate the CO_2_ Signaling Pathway

MPK12 belongs to the same group of MPKs as MPK4, a crucial regulator of pathogen and stress responses [29]. In tobacco, the silencing of MPK4 impaired CO_2_-induced stomatal closure [30]. Since *Arabidopsis* MPK4 and MPK12 are highly similar [31], it is possible that both MPK4 and MPK12 could regulate stomatal CO_2_ responses. Indeed, in an Y2H screen to identify HT1 interacting proteins, one prominent interactor was MPK4 [18].

**Fig 6:**
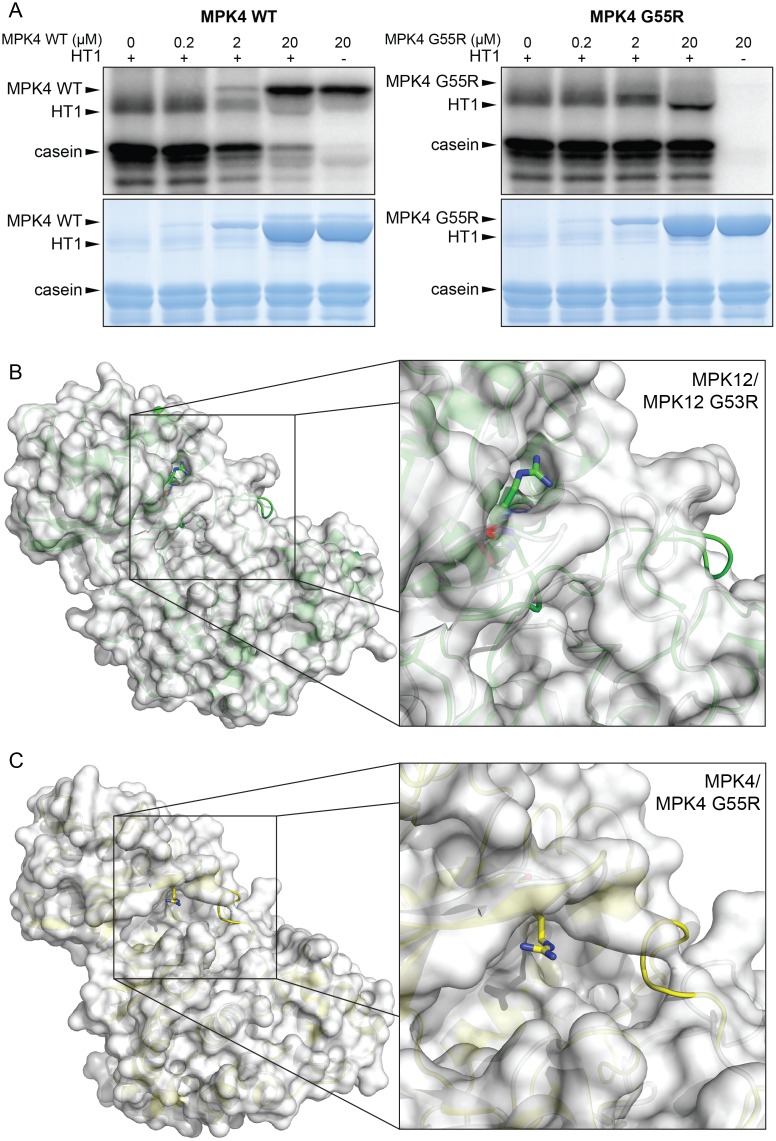
A conserved glycine is important for MPK4 and MPK12 function. (A) Inhibition of HT1 kinase activity in vitro by MPK4 and MPK4 G55R. Upper panel: autoradiography of the SDS PAGE gel; lower panel: Coomassie-stained SDS PAGE. Reaction mixture was incubated for 30 min. (B) Whole protein (left) and close-up (right) view of the superposition of models for MPK12 wild-type (secondary structure and surface in white) and MPK12 G53R (secondary structure in green). There is a close structural similarity between the structures except where the arginine at position 53 protrudes from the mutant protein surface and changes the loop region for the mutant. (C) Whole protein (left) and close-up (right) view of the superposition of models for MPK4 wild-type (secondary structure and surface in white) and MPK4 G55R (secondary structure in yellow). Similar to MPK12 G53R, the arginine at position 55 in MPK4 protrudes from the mutant protein surface and changes the loop region.

The *Arabidopsis mpk4* mutant is severely dwarfed, and measurements of accurate stomatal conductance with these plants are not feasible [32]. However, the impaired stomatal response to CO_2_ in *mpk12-4* (Fig 3) was further enhanced by guard cell–specific silencing of *MPK4* [18]; hence, in guard cells MPK4 is acting redundantly with MPK12 in stomatal CO_2_ signaling. Furthermore, MPK4 could also inhibit HT1 kinase activity [18]. The G53 residue in MPK12 is conserved in all *Arabidopsis* MPKs [10]. Since the G53R mutation blocked MPK12 function, we tested whether a similar mutation would impair MPK4 function. This experiment showed that MPK4-induced inhibition of HT1 activity was blocked by the introduction of a G55R mutation in MPK4; this mutation corresponds to G53R in Cvi-0 MPK12 (Fig 6A). Since MPK11 did not inhibit HT1 activity (S9 Fig), the function of MPKs as kinase inhibitors in *Arabidopsis* may be restricted to MPK12 and its closest homologue MPK4.

The *Arabidopsis* MPK6 crystal structure [33] was used to model the structure of MPK4 and MPK12 and to address the role of the G55R and G53R mutations that were shown to be crucial for the function of these proteins (Fig 6B and 6C). The mutation of Gly to Arg in position 53 in MPK12 caused the protrusion of the arginine sidechain on the surface of the protein, which could affect its binding affinity for other proteins in addition to an altered structure of the loop region. Similarly, the Arg in position 55 of MPK4 protruded from the surface as compared to the wild-type. Thus, the MPK12 G53R and MPK4 G55R amino acid substitutions may alter protein binding affinities of these MPKs to other proteins.

**Fig 7:**
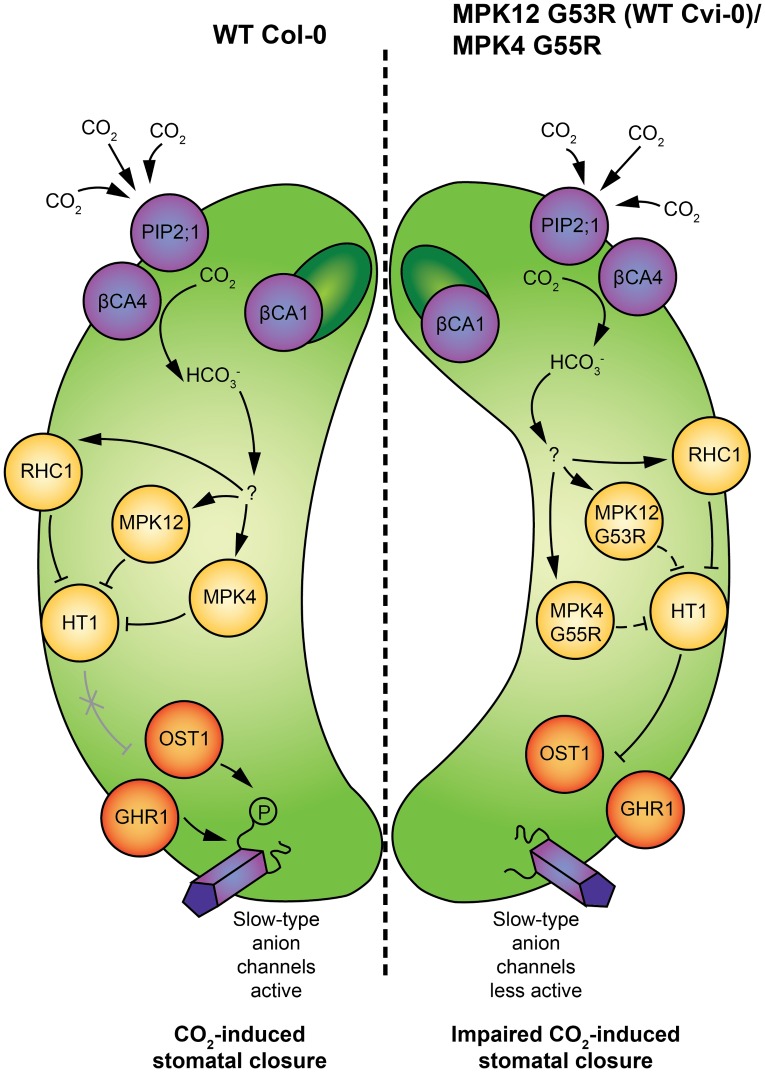
Schematic model of molecular events during elevated CO_2_-induced stomatal closure. (Left guard cell) CO_2_ enters guard cells through the PIP2;1 aquaporin [20] and is converted to bicarbonate by carbonic anhydrases βCA4 and βCA1. The mechanism by which bicarbonate is sensed in guard cells still needs to be resolved; nevertheless, it is likely that in elevated CO_2_ conditions, activation of MPK12 and MPK4 leads to inhibition of HT1, and this enables activation of slow-type anion channel SLAC1 by OST1 [15]. Additionally, GHR1 participates in the regulation of SLAC1 activity and is involved in CO_2_-induced stomatal closure [18]. Bicarbonate-induced inhibition of HT1 by RHC1 has also been shown [19]. (Right guard cell) The G53R mutation in Cvi-0 MPK12, as well as G55R mutation in MPK4, decreases the ability of these MPKs to inhibit HT1 kinase activity, which results in enhanced inhibition of SLAC1 activity by HT1 and decreased sensitivity to CO_2_ in stomatal closure.

Collectively, the presented experiments suggest that the CO_2_ signal leading to stomatal movements is transmitted through MPK12 and MPK4, leading to inhibition of HT1, and this enables SLAC1 activation by its activators, including OST1 and GHR1. Neither MPK12 G53R from Cvi-0 nor MPK4 G55R can fully inhibit HT1 (Fig 7).

## Discussion

Natural variation within a species holds great potential to identify regulatory mechanisms that are not easily uncovered through mutant screens. The Cvi-0 accession originates from the southern border of the *Arabidopsis* distribution area, the Cape Verde Islands. The Ler × Cvi RIL population was one of the first RILs produced, and it has been phenotyped for multiple traits [34]. Despite this, only a few QTLs from Cvi-0 have been identified at the molecular level. Our earlier research identified a locus related to ozone sensitivity and more open stomata phenotype of Cvi-0 in chromosome 2 [8]. Recently, the G53R substitution in MPK12 that affects plant water use efficiency was identified by using the Ler × Cvi populations, but the biochemical function of MPK12 in stomatal regulation was not further investigated [10]. Here, we generated NILs by backcrossing Cvi-0 eight times to Col-0 and show that the same natural mutation in Cvi-0 and lack of MPK12 in *cis* are the causes of ozone sensitivity, more open stomata, and altered CO_2_ responses of *Arabidopsis* plants. Furthermore, we showed that MPK12 regulates the activity of the protein kinase HT1, a major component of the CO_2_ signaling pathway in guard cells. The regulators of HT1 have remained largely unknown, despite the exceptionally strong CO_2_-insensitivity phenotype of plants with impaired HT1 function [12,15]. Our findings provide the first evidence for the role of MPK12 in guard cell CO_2_ signaling and provide a mechanistic insight for the MPK12 function in the regulation of plant water management.

The role of MPKs in *Arabidopsis* guard cell signaling has concentrated on MPK9 and MPK12, which are preferentially expressed in guard cells. Plants with point mutations in *MPK9* (*mpk9-1*, L295F) and *MPK12* (*mpk12-1*, T220I) had wild-type ABA responses, but *mpk12-1* has decreased WUE [10]. The *mpk9-1*, *mpk12-1*, and *mpk12-2* alleles are Tilling (Targeting Induced Local Lesions IN Genomes) lines in the Col-*erecta* background and the previously characterized MPK12-Cvi NIL is in the L*er* background [10,23]. Mutations in *ERECTA* modify transpiration efficiency and stomatal density, which may have influenced some of the previously described *mpk12-1* phenotypes [10,35]. In contrast, the full knockout alleles described here, *mpk12-3* and *mpk12-4*, are in Col-0 and imply a major function for MPK12 in CO_2_ signaling. Additional roles for MPK12 in stomatal responses have been inferred through the use of the double mutant *mpk9-1 mpk12-1* that has impaired stomatal closure responses to ABA and H_2_O_2_ treatment and has impaired S-type anion channel activation in response to ABA and Ca^2+^ [23]. It is also highly susceptible to *Pseudomonas syringae* infection and impaired in yeast elicitor-, chitosan-, and methyl jasmonate–induced stomatal closure [36]. Since the *mpk9 mpk12* double mutant appears to be more severely impaired in abiotic and biotic stomatal responses and S-type anion channel activation than the loss of function MPK12 alleles (Fig 3), it is possible that MPK12 together with MPK9 regulates stomatal aperture in response to various signals. MPK12 also regulates auxin responses in the root [11,26]. However, beyond the observation that plants with impaired MPK12 are hypersensitive to auxin inhibition of root growth, no details about the targets of MPK12 in roots are known.

HT1 was the first component shown to be specifically associated with stomatal CO_2_ signaling, and the *ht1-2* mutant has more closed stomata displaying constitutive high CO_2_ response at ambient CO_2_ levels (Fig 4D [12]). The opposite phenotypes of *mpk12* and *ht1-2* allowed us to use genetic analysis to position MPK12 in the guard cell signaling network. The stomata of *mpk12 ht1-2* were more closed, thus positioning MPK12 upstream of HT1 and possibly as a direct regulator of HT1 (Fig 4D). CO_2_ signaling in guard cells is initiated through the production of bicarbonate by carbonic anhydrases, and bicarbonate initiates signaling leading to activation of S-type anion channels [13,15]. In *mpk12*, the bicarbonate-dependent activation of S-type anion channels was impaired, as was previously found for the plants with impaired OST1 and SLAC1 (Fig 3E) [15]. The combined evidence from *mpk12* phenotypes, genetic analysis, and measurements of S-type anion currents all pointed towards MPK12 as a crucial regulator of CO_2_ signaling acting through HT1. Indeed, HT1 kinase activity was inhibited in the presence of Col-0 MPK12 but not by the Cvi-0 version of MPK12 (Fig 5A). Thus, the inhibitory function of MPK12 was impaired by the G53R amino acid substitution, probably by its weaker interaction with HT1 (Figs 4 and 5A). This explains the similar phenotypes of the NIL Col-S2, *mpk12-3*, and *mpk12-4*; they all display lack of inhibition of the negative regulator HT1, leading to higher stomatal conductance. Further support for the regulatory interplay between HT1 and MPK12 is provided by the isolation of a dominant mutation in *HT1*, *ht1*-*8D*, which in contrast to *ht1-2* has constitutively more open stomata and is biochemically resistant to inhibition by MPK12 [18]. Cvi-0 has altered phenotypes in many traits, including drought and pathogen resistance [34,37,38]. All of these traits are regulated through stomatal function; thus, the MPK12-HT1 regulatory module identified here may influence many of the previously observed phenotypes of Cvi-0.

Recently, two independent studies used *X*. *laevis* oocytes as a heterologous expression system to reconstitute bicarbonate-induced activation of the SLAC1 anion channel [19,20]. Tian et al. [19] reported that a multidrug and toxic compound extrusion (MATE)-type transporter RHC1 functions as a bicarbonate-sensing component that inactivates HT1 and promotes SLAC1 activation by OST1. More recently, it was demonstrated that expression of RHC1 alone was sufficient to activate ion currents in oocytes; these currents were independent of bicarbonate, calling into question the role of RHC1 as a bicarbonate sensor [20]. Furthermore, it was shown that SLAC1 activation can be reconstituted by extracellular bicarbonate in the presence of aquaporin PIP2;1, carbonic anhydrase CA4, and the protein kinases OST1, CPK6, and CPK23 [20]. However, in the guard cell, any proposed CO_2_ signaling pathway should include HT1, since plants with mutations in HT1 completely lack CO_2_-induced stomatal responses [12,18,28]. We showed that bicarbonate-induced S-type anion currents were strongly impaired in guard cell protoplasts, which lacked functional MPK12 (Fig 3E). Thus, MPK12, MPK4, and possibly other MPKs that are expressed in guard cells play a role in controlling the activity of HT1, and future research should identify the signaling pathway upstream of MPK12 (Fig 7). Dissection of different domains in SLAC1 revealed that the CO_2_ signal may involve the transmembrane region of SLAC1, whereas ABA activation of SLAC1 requires an intact N- and C-terminus [39]. Hence, ABA and CO_2_ regulation of SLAC1 could use different signaling pathways, and this may explain the lack of strong ABA phenotypes in plants with mutations in *MPK12*.

We propose that stomatal movements triggered by changes in CO_2_ concentration are regulated by MPK12- and MPK4-induced inhibition of HT1 activity (Fig 7). The MPK12 glycine 53 is conserved in all *Arabidopsis* MPKs [10] and is located on the protein surface in the glycine-rich loop that coordinates the gamma-phosphate of ATP (Fig 6B and 6C). Thus, this glycine may also be important for the function of other *Arabidopsis* MPKs. Further studies into the mechanisms controlling activation of MPKs in guard cells will help to identify molecular switches that function in plant acclimation to environmental stress and modulate the overall plant water use efficiency. Such information may allow the designing of molecular targets that can be used for breeding crops with improved water management.

## Materials and Methods

### Plant Material and Growth Conditions

Col-0, Col-*gl*, Cvi-0, *gdsl3-1* (GABI-492D11; CS447183), *cas-1* (SALK_070416), *cas-2* (GABI-665G12), and *cas-3* (SAIL_1157_C10) were from the European Arabidopsis Stock Centre (www.arabidopsis.info). Seeds of *ht1-2* were a gift from Dr. Koh Iba. Col-0 × Cvi-0 RILs were obtained from INRA Versailles. The *abi1-1* allele used was in Col-0 accession. Double mutants and other crosses were made through standard techniques and genotyped with PCR-based markers (S1 Table).

For ozone screening, seeds were sown at high density on a 1:1 v/v mixture of vermiculite and peat (type B2, Kekkilä, Finland), and kept for 2 d at 4°C for stratification. The plants were grown in controlled growth chambers (Bio 1300, Weiss Umwelttechnik, Germany) under a 12 h photoperiod, with a 23°C/19°C day/night temperature and a 70%/90% relative humidity or in growth rooms with equivalent growth conditions. The average photosynthetic photon flux density during the light period was 200 μmol m^-2^ s^-1^. When seedlings were 1 wk old, they were transplanted into 8 × 8 cm pots at a density of five plants per pot.

Three-week-old plants were exposed to ozone in growth chambers under the same conditions as they were grown until the experiments. Ozone exposure was acute (300–350 ppb for 6 h) and started 2 h after light was switched on. Ozone damage was visualized with trypan blue stain or quantified as electrolyte leakage.

### Mapping of Cvi-0 Ozone Sensitivity QTLs

NILs were created by crossing Col-0 with Cvi-0 and selecting the most ozone-sensitive plant in F2 and backcrossing to Col-0 for eight generations (generating Col-S) or selecting the most tolerant plant and backcrossing to Cvi-0 for six generations (generating Cvi-T). The genomes of Cvi-0 and Cvi-T were sequenced at BGI Tech Solutions (Hong Kong) with Illumina technology, and the genomes of Col-S and Cvi-T were sequenced at the DNA Sequencing and Genomics lab of the University of Helsinki with SOLiD technology. Genome sequence data is available from the NCBI BioProject database with the accession number PRJNA345097. The 90-bp-long Illumina paired end sequencing library reads were mapped onto the Col-0 reference genome (TAIR10) with using the Bowtie2 aligner (version 2.0.0-beta7; [40]) in “end-to-end” alignment mode, yielding an average genomic sequence coverage of 45-fold. Variation calling and haplotype phasing was performed with the help of samtools (tools for alignments in the SAM format, Version: 0.1.18; [41]). Based on the aligned sequences, various PCR-based markers (S1 Table) were designed to genotype Cvi-0 versus Col-0 in the NILs and informative RILs from the INRA Versailles Col-0 × Cvi-0 RIL population. The markers were also used to genotype ozone-sensitive individuals from segregating F2 populations.

### Mapping of *cis* Mutation

Mapping population was created by crossing *cis* (Col-0) and C24 as an *Arabidopsis* accession with low stomatal conductance. High water loss from excised leaves and decreased responses to high CO_2_ were used as a selective trait. Rough mapping with 22 markers using 59 F2 samples showed linkage to the bottom of chromosome 2, at the marker UPSC_2–18415 at 18.4 Mbp. Pooled genomic DNA from 66 selected F3 plants was used for sequencing. Whole genome sequencing was conducted with Illumina HiSeq 2000, and the reads were mapped against Col-0 genome (release TAIR10) by BGI Tech Solutions (Hong Kong). Genome sequence data is available from the NCBI BioProject database with the accession number PRJNA345097 and PRJNA343292. For mapping the genomic area of the mutation, the Next Generation Mapping tool was used [42], which positioned the mutation on chromosome 2 between 18,703,644 –19,136,098 bp. The deletion mutation in *cis* was verified by PCR to be 4,770 bp (at the position 18,945,427–18,950,196 bp).

### Complementation Lines

*MPK12* and its promoter were amplified from Col-0 or Cvi-0 genomic DNA using Phusion (Thermo Fisher Scientific) and Gateway (Invitrogen) cloned into entry vector pDONR-Zeo. Subsequently, the genes were cloned into pGWB13 and pMCD100. Plants were transformed with floral dipping [43].

### Southern Blotting Analyses

Total DNAs from different genotyping plants were extracted by CTAB method, and 12 micrograms of total DNA was digested by HindIII or EcoRI. The DNAs were running on the gel and transformed onto Nylon membrane. Hybridization was performed with digoxigenin-labeled specific genomic DNA amplified by primers F3 and R4 for 12 h. The membrane was washed several times by washing buffer and Maleic acid buffer. The membrane was blocked by blocking solution for 1 h at room temperature and washed and incubated with anti-DIG-AP for 30 min. Detection was performed using substrate DIG CSPD.

### Plant Growth and Experimental Settings for Gas Exchange Measurements

Seeds were planted on a soil mixture consisting of 2:1 (v:v) peat:vermiculite and grown through a hole in a glass plate covering the pot as described previously [44]. Plants were grown in growth chambers (MCA1600, Snijders Scientific, Drogenbos, Belgium) at 12 h/12 h day/night cycle, 23°C/20°C temperature, 100 μmol m^-2^ s^-1^ light, and 70% relative humidity (RH). For gas exchange experiments, 24- to 30-d-old plants were used.

Stomatal conductance of intact plants was measured using a rapid-response gas exchange measurement device consisting of eight through-flow whole-rosette cuvettes [44]. The unit of stomatal conductance mmol m^-2^ s^-1^ reflects the amount of H_2_O moles that exits the plant through stomata per one m^2^ of leaf area per second. Prior to the experiment, plants were acclimated in the measurement cuvettes in ambient CO_2_ concentration (~400 ppm), 100 μmol m^-2^ s^-1^ light (if not stated otherwise), and ambient humidity (RH 65%–80%) for at least 1 h or until stomatal conductance was stable. Thereafter, the following stimuli were applied: decrease or increase in CO_2_ concentration, darkness, reduced air humidity, and ozone. CO_2_ concentration was decreased to 100 ppm by filtering air through a column of granular potassium hydroxide. In CO_2_ enrichment experiments, CO_2_ was increased by adding it to the air inlet to achieve a concentration of 800 ppm. Darkness was applied by covering the measurement cuvettes. In blue light experiments, dark-adapted plants were exposed to blue light (50 μmol m^-2^ s^-1^) from an LED light source (B42180, Seoul Semiconductor, Ansan, South Korea). The decreased or increased CO_2_ concentration, darkness, and blue light were applied for 58 min. In the long-term elevated CO_2_ experiment (Fig 1D and S1E Fig), CO_2_ concentration was increased from 400 ppm to 800 ppm for 2.5 h. To calculate stomatal half-response time, the whole 2.5-h stomatal response to elevated CO_2_ was scaled to a range from 0% to 100%, and the time when 50% of stomatal closure had occurred was calculated. Humidity was decreased by a thermostat system to 30%–40% RH, and stomatal conductance was monitored for another 56 min. In ozone experiments, the plants were exposed to 350–450 ppb of ozone for 3 min and stomatal conductance was measured for 60 min after the start of the exposure.

In ABA-induced stomatal closure experiments, 5 μM ABA solution was applied by spraying as described in [45]. At time point 0, plants were removed from cuvettes and sprayed with either 5 μM ABA solution (5 μM ABA, 0.012% Silwet L-77 [PhytoTechnology Laboratories], and 0.05% ethanol) or control solution (0.012% Silwet L-77 and 0.05% ethanol). Thereafter, plants were returned to the cuvettes and stomatal conductance was monitored for 56 min.

In ABA-induced inhibition of stomatal opening experiments, plants were acclimated in measurement cuvettes in darkness. At time point 0, plants were removed from cuvettes and sprayed with 2.5 μM ABA solution (2.5 μM ABA, 0.012% Silwet L-77 [PhytoTechnology Laboratories], and 0.05% ethanol) or control solution (0.012% Silwet L-77 and 0.05% ethanol). Thereafter, plants were returned to the cuvettes, dark covers were removed, and stomatal conductance was monitored in light for 56 min.

Prior to the measurement of the diurnal pattern of stomatal conductance, plants were preincubated in the measurement cuvette for at least 12 h in respective light and humidity conditions. Plants were measured in 16-min intervals. WUE was calculated based on the data of diurnal experiments as an average of daytime light period (from 9:00 to 17:00).

CO_2_-induced stomatal conductance in S2 Fig was measured as following. Five-week-old healthy plants growing in a growth chamber with 70% humidity and a 16 h light/8 h dark condition were used for stomatal conductance analyses at different CO_2_ concentrations by a LiCOR-6400XT, as previously described [13]. Relative stomatal conductance values were normalized relative to the last data point preceding the [CO_2_] transitions (400 to 800 or 1,000 ppm).

### Stomatal Aperture

The *MPK12* deletion mutant *mpk12-4* and wild-type plants were grown in a growth chamber at 70% humidity, 75 μmolm^-2^ s^-1^ light intensity, 21°C, and 16 h light/8 h dark regime. Leaf epidermal layers from 2-wk-old plants of both genotypes were preincubated in an opening buffer (10 mM MES, 10 mM KCl, and 50 mM CaCl_2_ at pH 6.15) for 2 h, and stomata were individually imaged and tracked for measurement before treatment. After that, the leaf epidermal layers were incubated with buffers containing 10 μM ABA for 30 min and the individually tracked stomata were imaged. Stomatal apertures were measured by ImageJ software and genotype-blind analyses were used. The data presented are means and SEM *n* = 3 experiments, with 30 stomata per experiment and condition.

### Stomatal Index and Density

Plants at the age of 28–30 d were used for stomatal index and density measurements. Rosette leaves of equal size were excised, and the abaxial side was covered with the dental resin (Xantopren M mucosa, Heraeus Kulzer, Germany). Transparent nail varnish was applied onto the dried impressions after the removal of the leaves. The hardened nail varnish imprints were attached onto a microscope glass slide with a transparent tape and imaged under a Zeiss SteREO Discovery.V20 stereomicroscope. For quantification, an image with the coverage of 0.12 mm^2^ was taken from the middle of the leaf, next to the middle vein. In total, 81–84 plants per line from two independent biological repeats were analyzed—one leaf from each plant, one image from each leaf. Stomatal index was calculated with the following formula: SI = Stomatal density / (Density of other epidermal cells + Stomatal density).

### Stomatal Complex Length

For the stomatal complex length measurements, plants at the age of 28–35 d were used. Whole leaves were preincubated for 4 h abaxial side down in the buffer (10 mM MES, 5 mM KCl, 50 μM CaCl_2_, pH 6.15 [with TRIS]) in the light. Four to six plants per genotype and one leaf per plant were analyzed, and altogether 84–126 stomatal complexes per genotype were measured.

### Y2H Interaction Tests

Interactions between MPK12 and selected protein kinases and phosphatases were tested in pairwise split-ubiquitin Y2H assays using the DUALhunter and DUALmembrane 3 kits (Dualsystems Biotech). For bait construction, the coding sequences of *MPK12* were PCR-amplified from total cDNAs from Col-0 and Cvi-0. Other *MPK12* variants with point mutations (K70R, Y122C, and D196G+E200A) were created by two-step overlap PCR using the Col-0 *MPK12* as a template. *HT1* was also PCR-amplified from Col-0 cDNA. All *MPK12*s and *HT1* were digested with SfiI and cloned to the corresponding site in pDHB1, which contained the Cub-LexA-VP16 fusion. For prey constructs, coding sequences of each selected gene were amplified from total Col-0 cDNAs, digested with SfiI, and cloned into either pPR3-N (*HT1*, *OST1*, *BLUS1*, *IBR5*, *MKP2*, *MPK12*, *MPK12G53R*, *MPK11*) or pPR3-STE (*SnRK2*.*2*, *SnRK3*.*11*, *ABI1*, *ABI2*, *HAB1*, *HAB2*), which contained a mutated NubG. All primers used are listed in Table S1. The pAI-Alg5 with a native NubI was used as a positive prey control, whereas the pDL2-Alg5 containing NubG served as a negative control.

For pairwise Y2H assays, the yeast strain NMY51 was cotransformed with bait and prey plasmids and grown on SD-Leu-Trp plates to select for presence of both plasmids. At least ten colonies from each transformation were pooled and resuspended in water to an OD600 of 0.5, from which 100, 1,000, and 10,000x serial dilutions were prepared and spotted on SD-Leu-Trp and SD-Leu-Trp-His-Ade plates. SD-Leu-Trp plates were incubated at 30°C for 2 d, photographed, and used for β-galatosidase overlay assays. SD-Leu-Trp-His-Ade plates were incubated for 2–4 d and photographed. The quantitative β-galactosidase assay was performed with three pools of ten independent colonies from each pairwise combination using the Yeast β-galactosidase assay kit (Thermo Fisher Scientific) by the nonstop quantitative method.

### Ratiometric BiFC Assay

Binary constructs containing split YFPs were designed and generated for cloning genes of interest by the ligation independent cloning (LIC) method as described in [18].

Each gene of interest was amplified by two consecutive PCR reactions: first with gene-specific primers and later with a pair of universal primers designed specifically for the LIC method. All primers used are listed in S1 Table. To prepare vectors for LIC, plasmids of 35S:YFPn and 35S:YFPc were linearized by PmlI digestion, followed by T4 DNA polymerase treatment with dGTP to create 15–16 nucleotide 5ʹ-overhangs. For insert preparation, the final PCR products of target genes were incubated with T4 DNA polymerase in the presence of dCTP to create the complementary overhangs with the vectors. Both vector and insert were mixed at room temperature and proceeded with *Escherichia coli* transformation after 5 min. The final constructs were sequence verified and transformed to *Agrobacterium tumefaciens* GV3101 for agro-infiltration experiments.

For the ratiometric BiFC assays, four different agrobacterial clones—each harboring a YFPn fusion, a YFPc fusion, the SLAC1-CFP internal control, or the gene silencing suppressor P19—were co-infiltrated to the leaves of *N*. *benthamiana* at an OD600 of 0.02 for each clone in the infiltration buffer (10mM MES, 10mM MgCl_2_, 200 μM acetosyringone). Images were acquired at 3 dpi with a Zeiss LSM710 confocal microscope using a 63x objective (for high magnification images) or a 20x objective (for fluorescence quantification). The YFP signals were excited by a 514 nm laser, and emission between 518–564 nm was collected. The CFP signals were excited by a 405 nm laser, and emission at 460–530 was collected. Z-stack images of approximately 15 μm thickness were collected, and all images were acquired at the 16-bit depth for a higher dynamic range. The fluorescence intensity was measured by the ImageJ software. The leaf samples used for imaging were collected and used for protein extraction followed by western blot analysis.

### Western Blot Analysis

The leaf samples (30–40 mg) were ground under liquid nitrogen and boiled for 10 min in 100 μL of 6X Laemmli buffer. 12 μL of each sample were separated on 10% SDS polyacrylamide gel. After SDS-PAGE, proteins were transferred onto nitrocellulose membrane. Immunodetection of HA-tagged proteins was performed with a monoclonal anti-HA antibody.

### Split Luciferase Complementation Assay

The *MPK12* cDNA was cloned into a vector containing the N-terminal half of luciferase (nLUC) and *HT1* was cloned into the cLUC. The constructs in the *A*. *tumefaciens* strain GV3101 were co-infiltrated into *N*. *benthamiana* leaves with P19 at an OD600 of 0.8. The infiltrated leaves after 3 d of infiltration were harvested for bioluminescence detection. Images were captured with a CCD camera.

### Measurement of S-type Anion Currents

*Arabidopsis* guard cell protoplasts were isolated as described previously [46]. Guard cell protoplasts were washed twice with a washing solution containing 1 mM MgCl_2_, 1 mM CaCl_2_, 5 mM MES, and 500 mM D-sorbitol (pH 5.6 with Tris). During patch clamp recordings of S-type anion currents, the membrane voltage started at +35 to –145 mV for 7 s with –30 mV decrements, and the holding potential was +30 mV. The bath solutions contained 30 mM CsCl, 2 mM MgCl_2_, 10 mM MES (Tris, pH 5.6), and 1 mM CaCl_2_, with an osmolality of 485 mmol/kg. The pipette solutions contained 5.86 mM CaCl_2_, 6.7 mM EGTA, 2 mM MgCl_2_, 10 mM Hepes-Tris (pH 7.1), and 150 mM CsCl, with an osmolality of 500 mmol/kg. The free calcium concentration was 2 μM. The final osmolalities in both bath and pipette solutions were adjusted with D-sorbitol. Mg-ATP (5 mM) was added to the pipette solution before use. 13.5 mM CsHCO_3_ (11.5 mM free [HCO_3_^-^] and 2 mM free [CO_2_]) was freshly dissolved in the pipette solution before patch clamp experiments. The concentrations of free bicarbonate and free CO_2_ were calculated using the Henderson–Hasselbalch equation (pH = pK1 + log [HCO_3_^-^] / [CO_2_]). pK1 = 6.352 was used for the calculation. [HCO_3_^-^] represents the free bicarbonate concentration and [CO_2_] represents the free CO_2_ concentration.

### Protein Expression and Purification

For in vitro kinase assays, the respective sequences of HT1, HT1 K113M, MPK11, MPK12, MPK12 G53R, MPK12 K70R, and MPK12 Y122C were cloned into a pET28a vector (Novagen, Merck Millipore) using primers listed in S1 Table. Point mutations corresponding to K113M in HT1, K70R in MPK12, and Y122C in MPK12 were created with two-step PCR using primers listed in S1 Table. *MPK4* was cloned as previously described [18].

6xHis-HT1WT, 6xHis-HT1 K113M, 6xHis-MPK12, 6xHis-MPK12 G53R, 6xHis-MPK12 K70R, 6xHis-MPK12 Y122C, 6xHis-MPK11, 6xHis-MPK4 WT, and 6xHis-MPK4 G55R were expressed in *E*. *coli* BL21(DE3) cells. A 2 mL aliquot of an overnight culture was transferred to a fresh 1 L 2xYT medium and grown at 37°C to an absorbance of ~0.6 at OD600. The cultures were chilled to 16°C and recombinant protein expression was induced by 0.3 mM isopropyl b-D-thiogalactopyranoside for 16 h. The cells were harvested by centrifugation (5,000 rpm, 10 min, 4°C) and stored at –80°C until use.

All purification procedures were carried out at 4°C. The cells were resuspended in 30 mL of lysis buffer (50 mM Tris-HCl [pH 7.4], 300 mM NaCl, 5% [v/v] glycerol, 1% [v/v] Triton X-100, 1 mM PMSF, 1 μg/ml aprotinin, 1 μg/ml pepstatin A, 1 μg/ml leupeptin) and lysed using an Emulsiflex C3 Homogenizer. Cell debris was removed by centrifugation at 20,000 rpm for 30 min. The protein-containing supernatant was mixed for 1 h at 4°C with 0.20 mL of Chelating Sepharose Fast Flow resin (GE Healthcare), charged with 200 mM NiSO_4_ and pre-equilibrated in the lysis buffer. The protein–resin complex was packed into a column, and the beads were washed with 5x10 column volumes (CV) of a wash buffer I (50 mM Tris-HCl [pH 7.4], 600 mM NaCl, 5% [v/v] glycerol, 1% [v/v] Triton X-100), 5x10 CV of a wash buffer II (50 mM Tris-HCl [pH 7.4], 300 mM NaCl, 5% [v/v] glycerol, 0.1% [v/v] NP-40), and 2x10 CV of a wash buffer III (50 mM Tris-HCl [pH 7.4], 150 mM NaCl, 5% [v/v] glycerol, 0.1% [v/v] NP-40). The protein was eluted by incubating the beads for 5 min at room temperature with an imidazole-containing elution buffer (50 mM Tris-HCl, 150 mM NaCl, 5% [v/v] glycerol, 0.1% [v/v] NP-40, 300 mM imidazole). MPK12 proteins were concentrated and imidazole was removed by Millipore Amicon Ultra-0.5 Centrifugal Filter Concentrators (NMWL 3000). Glycerol was added to a final concentration of 20% (v/v), and 20 μL aliquots of the eluted protein were snap-frozen in liquid nitrogen and stored at –80°C.

### In Vitro Kinase Assays

Protein concentrations were estimated on 10% SDS-polyacrylamide gel using BSA as a standard. HT1 kinase activity assay was performed by incubating a constant amount of purified recombinant HT1 and 0–30 μM MPK12, 0–20 μM MPK4, or 0–10 μM MPK11 in a reaction buffer (50 mM Tris-HCl [pH 7.4], 150 mM NaCl, 20 mM MgCl2, 60 mM imidazole, 1 mM DTT, 0.2 mg/ml insulin) at room temperature for 10 min. Then, casein (1 mg/ml), 500 μM ATP, and 100 μCi/ml 32P-γ-ATP were added and reaction aliquots were taken at the 30 min time point. Reactions were stopped by the addition of SDS loading buffer. Proteins were separated on a 10% SDS-polyacrylamide gel and visualized by Coomassie brilliant blue R-250 (Sigma) staining. HT1 activity was determined by autoradiography and quantified by ImageQuant TL Software.

### Model of MPK12 and MPK4

Sequence searches and alignments were conducted with SWISS-MODEL [47]. The crystal structure with the best sequence identity and resolution was selected for building homology models. *Arabidopsis* MPK12 and MPK4 have sequence identity to the 3 Ångstrom resolution *Arabidopsis* MPK6 structure (5CI6; [33]) of 64.61% and 70.67%, respectively. This structure was then used to construct models for the wild-type and mutant structures. The RMSD from aligning the structures for MPK12 and MPK12 G53R was 0.324 Ångstroms (i.e., a close structural similarity). Structures were checked for clashes and with quality controls and were then superposed.

### Statistical Analysis

Statistical analyses were performed with Statistica, version 7.1 (StatSoft Inc., Tulsa, Oklahoma, United States). All effects were considered significant at *p* < 0.05.

## Supporting Information

S1 Fig Identification of Col-S2 and *cis* mutation. (A) Ion leakage after 6 h of ozone exposure (350 ppb ozone). Experiment was repeated three times (mean ± SD; 1-way ANOVA of ozone treated plants). (B) Scheme of mapping the ozone sensitive trait of Col-S2. (C) CO_2_-induced changes in stomatal conductance in *cas* mutants (mean ± SEM; n = 5–6 plants). (D) Mapping scheme of *cis* mutation obtained from *cas-2* T-DNA line. (E) Stomatal response to elevation of the atmospheric CO_2_ concentration from 400 ppm to 800 ppm at time point 0. Data are given as average stomatal conductance, ± SEM of Col-0 (n = 13), Col-S2 (n = 13) and *mpk12-4* (n = 13). The data were pooled from two experimental series. (F) Stomatal conductance of Col-0 plants transformed with MPK12-Cvi in T1 generation (mean ± SEM; 1-way ANOVA, Tukey HSD post hoc test for unequal sample size; n = 4–16 plants). (G) Stomatal conductance of F1 generation of Col-S2 x *gl1* (mean ± SEM; 1-way ANOVA, Tukey HSD post hoc test for unequal sample size). Experiment was repeated two times (n = 10–60 plants). The raw data for panels (A), (C), (E-G) can be found in S1 Data file.
(TIF)

S2 Fig Mutations in *MPK12* are causing impaired CO_2_-responses in both *cas-2* and *gdsl3-1* mutants. (A) The originally described T-DNA insertions were confirmed in *cas-2* and *gdsl3-1* (GABI_492D11) plants by genotyping analyses. PCR product for the *CAS* gene was amplified by primers CASLP and CASRP. PCR product for the *cas-2* insert was amplified by primers CASRP and GABILb. PCR product for the *GDSL3* gene was amplified by primers GDSL3RP and GDSL3LP. PCR product for the *gdsl3-1* insert was amplified by primers GDSL3LP and GABILb. Lane 9: DNA marker. (B) Time-resolved relative stomatal conductance analyses showed that CO_2_-induced stomatal closing was greatly impaired in *CAS* mutant allele *cas-2*, but not in *cas-1* allele. Data present are means ± SEM, n = 3 leaves for wild type and n = 4 leaves for *CAS* alleles. (C) Time-resolved relative stomatal conductance analyses showed that CO_2_-induced stomatal closure was greatly impaired in *gdsl3-1*. Data present are means ± SEM, n = 3 leaves for each genotype. (D) Thermal imaging showed that *cas-2* and *gdsl3-1* have much lower leaf temperature compared to *cas-1* and Col-0 plants. (E, F) Southern blotting confirmed that a 4770 bp region containing *MPK12* and *BYPASS2* between R3 and F21 was deleted in *cas-2* and *gdsl3-1* mutants. Genomic DNAs extracted from *cas-2*, *gdsl3-1* and Col-0 were digested with HindIII and BamHI. The probe was set in the region F3 and F2 marked in E. (G) *mpk12-4* mutant from *gdsl3-1*×Col-0 backcross F2 offsprings, in which *MPK12-BYPASS2* was deleted but contained *GDSL3*, displayed similar responses to CO_2_ changes as *gdsl3-1* by gas exchange analyses. Data present are means ± SEM, n = 3 leaves for each genotype. (H) Time-resolved relative stomatal conductance analyses showed that expression of *MPK12* under the control of *UBQ10* promoter in *gdsl3-1* complemented the insensitive stomatal CO_2_ responses. Data present are means ± SEM, n = 3 leaves for each genotype. The raw data for panels (B-C), (G-H) can be found in S1 Data file.
(TIF)

S3 Fig RT-PCR analysis of full length *MPK12* transcript in Col-0, *mpk12-3*, and *mpk12-4* plants. *ACTIN2* was amplified as a control.
(TIF)

S4 Fig Stomatal index, length, and density in *mpk12*. (A) Stomatal index of studied lines (mean ± SEM; 1-way ANOVA, Tukey HSD post hoc test). Experiment was repeated twice (n = 81–84 plants). (B) Stomatal complex length of *mpk12* lines (mean ± SEM; 1-way ANOVA). Sample size was 4–6 plants, altogether 84–126 stomatal complexes per line were measured. (C) Stomatal density of studied lines (mean ± SEM; 1-way ANOVA). Experiment was repeated twice (n = 81–84 plants). The raw data for panels (A-C) can be found in S1 Data file.
(TIF)

S5 Fig Time-dependent changes in stomatal conductance. Various stimuli were applied as indicated by the bars or arrows in the legends of each panel. Stomatal opening induced by 100 ppm CO_2_ (A) and 50 μmol m^-2^s^-1^ blue light (B). ABA inhibited light-induced stomatal opening (C). Stomatal closure in response to darkness (D), 800 ppm CO_2_ (E), decrease in air humidity (F), a 3-minute O_3_ pulse (G) and spraying the rosette with 5 μM ABA solution (H). The data in all the figures is represented as mean ± SEM. All experiments were repeated at least three times (n = 11–18). The raw data for panels (A-H) can be found in S1 Data file.
(TIF)

S6 Fig Deletion of *MPK12* did not affect ABA-induced stomatal closure. The stomata in the *MPK12* deletion mutant *mpk12-4* closed after treatment with 10 μM ABA for 30 min, similar as in wild type. Data are average of 3 experiments, 10 stomata per experiment and condition. Small letters denote statistically significant differences according to 2-way ANOVA with Tukey HSD post hoc test. The raw data for the figure can be found in S1 Data file.
(TIF)

S7 Fig MPK12 interacts with HT1 and IBR5. Split-ubiquitin yeast two-hybrid assays with MPK12 and different versions of MPK12 with amino acid substitutions; MPK12 G53R with the same point mutation as in Cvi-0, MPK12 K70R kinase inactive version, MPK12 Y122C and MPK12 D196G, E200A constitutively active kinase versions. (A) Yeast growth observed on SD-leu-trp plate without 3-amino-1,2,4-triazole (3-AT), 24 hours of X-Gal incubation. (B) Yeast growth observed on SD-leu-trp-his-ade plate with 20 mM 3-AT. (C) Split luciferase complementation assays showed that MPK12 interacts with HT1 in tobacco leaves. MPK12:nLUC with only cLUC was used as negative control, and showed no luciferase bioluminescence signal.
(TIF)

S8 Fig Stable expression of YFP-labelled MPK12 in intact leaves of *Arabidopsis thaliana* and transient expression in leaves of *Nicotiana benthamiana*. Expression of MPK12-YFP (A) and MPK12 G53R-YFP (B) under native *MPK12* promoter in *A*. *thaliana* Col-0. Transient expression under the CaMV35S promoter was also shown for MPK12-YFP (C) and MPK12 G53R-YFP in *N*. *bethamiana* (D). Scale bar = 50 μm.
(TIF)

S9 Fig MPK11 does not inhibit the activity of HT1. MPK11, an MPK from the same group as MPK12, was not able to inhibit HT1 showing that not all the Arabidopsis MPKs are inhibitors of HT1. This experiment was repeated four times.
(TIF)

S1 Table Primers used in this study. (DOCX)

S1 Data Raw data for all figures and supplemental figures. (XLSX)

S1 Video A time course of Col-0, Cvi-0, Col-S and Cvi-T exposed to 350 ppb ozone. (WMV)

## Abbreviations

ABA: abscisic acid
*abi1-1*: *ABA insensitive 1–1*
BiFC: bimolecular fluorescence complementation
CA4: CARBONIC ANHYDRASE4
CAS: calcium-sensing receptor
GHR1: guard cell hydrogen peroxide-resistant1
HSD: honest significant difference
HT1: high leaf temperature1
IBR5: indole-3-butyric acid response5
MAP: mitogen-activated protein
MATE: multidrug and toxic compound extrusion
MPK12: mitogen-activated protein kinase 12
NIL: near-isogenic line
OST1: open stomata1
QTL: quantitative trait loci
RHC1: resistant to high carbon dioxide1
RIL: recombinant inbred line
SAIL: Syngenta Arabidopsis Insertion Library
SLAC1: slow anion channel1
T-DNA: transfer DNA
TILLING: Targeting Induced Local Lesions IN Genomes
WUE: water use efficiency
Y2H: yeast two-hybrid
