# Supplementary figures and images for: Natural Variation in Arabidopsis Cvi-0 Accession Reveals an Important Role of MPK12 in Guard Cell CO2 Signaling
Source: PLoS Biol. 2016 Dec 6;14(12):e2000322. doi: 10.1371/journal.pbio.2000322 (PMC5147794; doi:10.1371/journal.pbio.2000322)

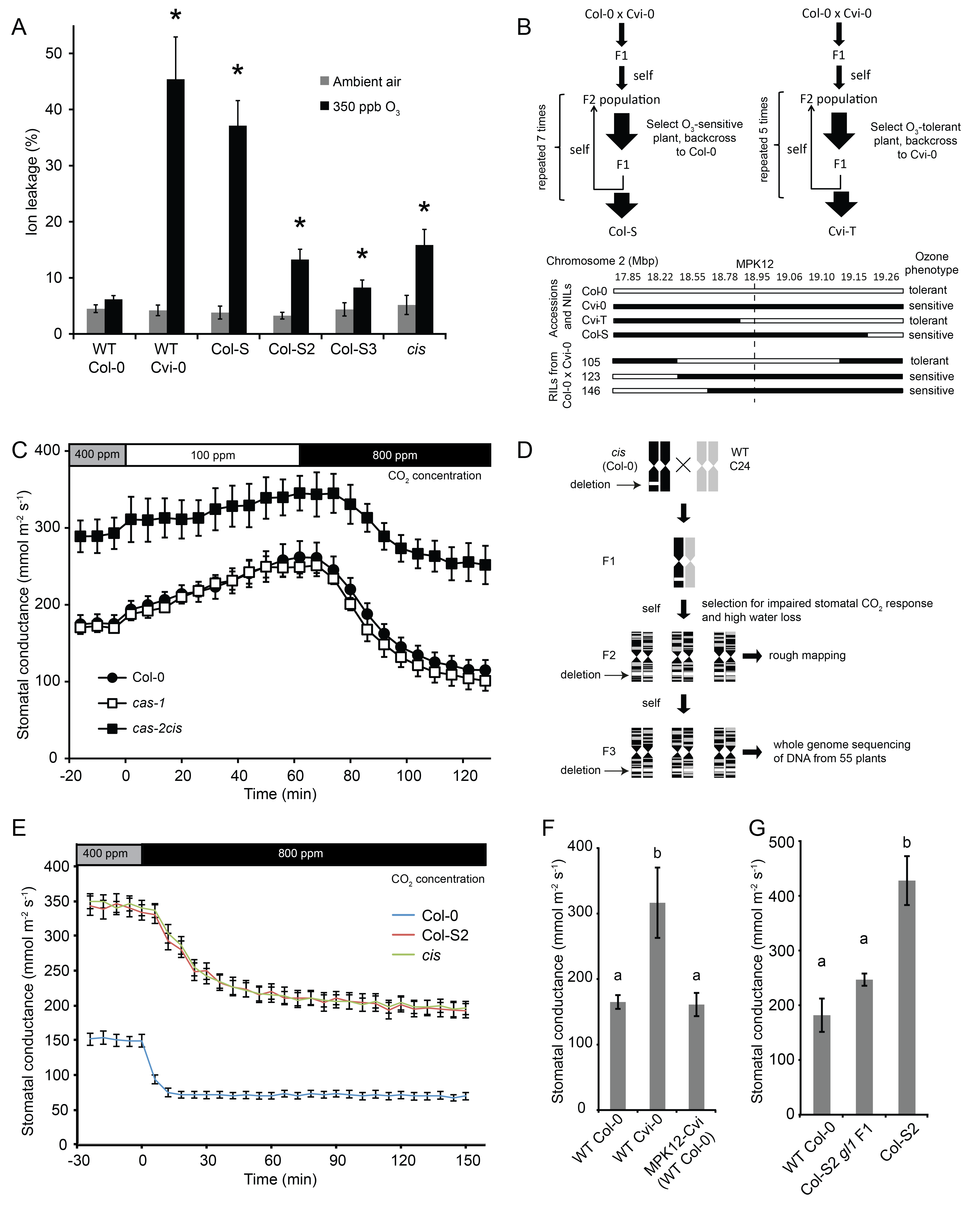

Supplement: S1 Fig — (A) Ion leakage after 6 h of ozone exposure (350 ppb ozone). Experiment was repeated three times (mean ± SD; 1-way ANOVA of ozone treated plants). (B) Scheme of mapping the ozone sensitive trait of Col-S2. (C) CO2-induced changes in stomatal conductance in cas mutants (mean ± SEM; n = 5–6 plants). (D) Mapping scheme of cis mutation obtained from cas-2 T-DNA line. (E) Stomatal response to elevation of the atmospheric CO2 concentration from 400 ppm to 800 ppm at time point 0. Data are given as average stomatal conductance, ± SEM of Col-0 (n = 13), Col-S2 (n = 13) and mpk12-4 (n = 13). The data were pooled from two experimental series. (F) Stomatal conductance of Col-0 plants transformed with MPK12-Cvi in T1 generation (mean ± SEM; 1-way ANOVA, Tukey HSD post hoc test for unequal sample size; n = 4–16 plants). (G) Stomatal conductance of F1 generation of Col-S2 x gl1 (mean ± SEM; 1-way ANOVA, Tukey HSD post hoc test for unequal sample size). Experiment was repeated two times (n = 10–60 plants). The raw data for panels (A), (C), (E-G) can be found in S1 Data file. (TIF) [file pbio.2000322.s001.tif]

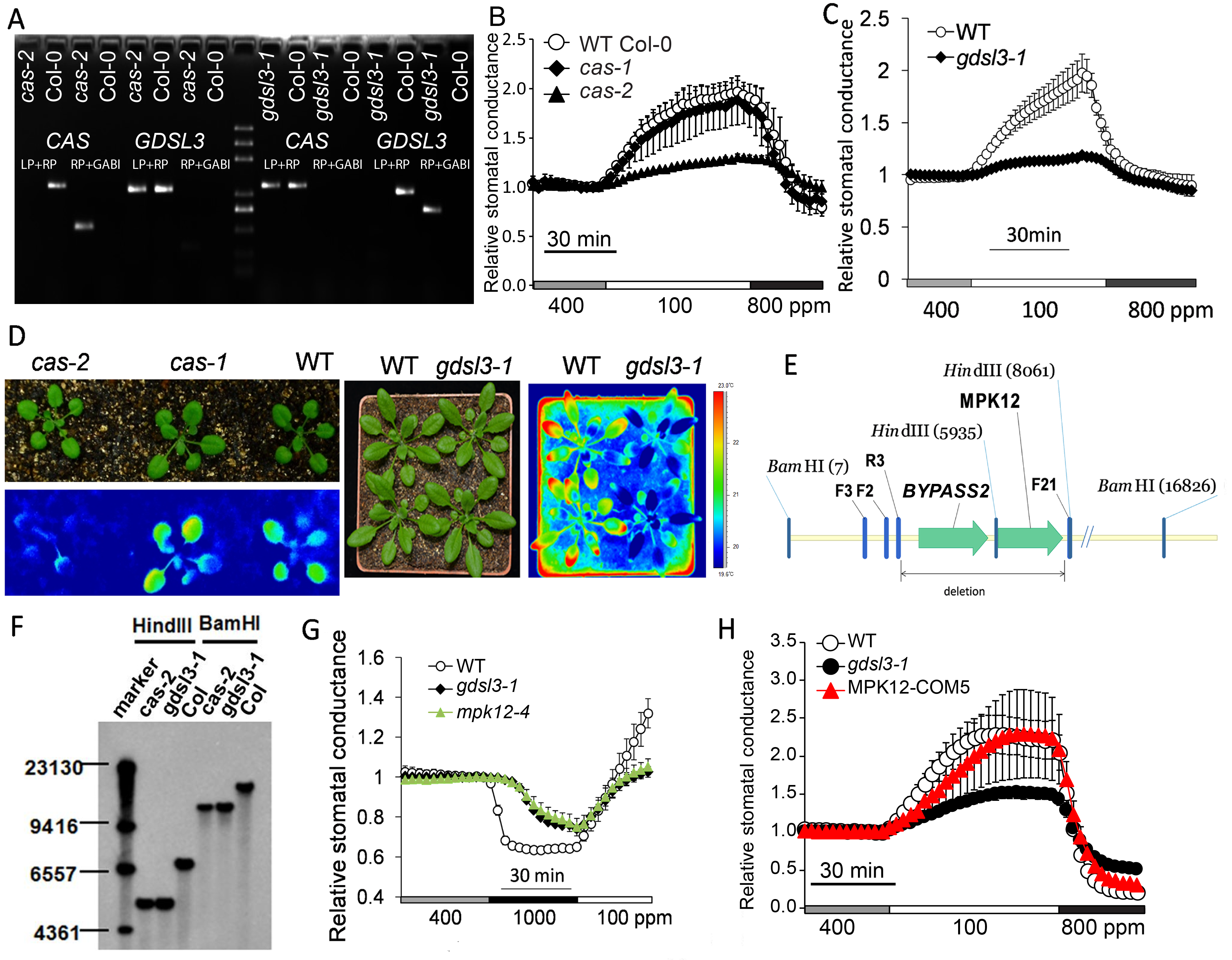

Supplement: S2 Fig — (A) The originally described T-DNA insertions were confirmed in cas-2 and gdsl3-1 (GABI_492D11) plants by genotyping analyses. PCR product for the CAS gene was amplified by primers CASLP and CASRP. PCR product for the cas-2 insert was amplified by primers CASRP and GABILb. PCR product for the GDSL3 gene was amplified by primers GDSL3RP and GDSL3LP. PCR product for the gdsl3-1 insert was amplified by primers GDSL3LP and GABILb. Lane 9: DNA marker. (B) Time-resolved relative stomatal conductance analyses showed that CO2-induced stomatal closing was greatly impaired in CAS mutant allele cas-2, but not in cas-1 allele. Data present are means ± SEM, n = 3 leaves for wild type and n = 4 leaves for CAS alleles. (C) Time-resolved relative stomatal conductance analyses showed that CO2-induced stomatal closure was greatly impaired in gdsl3-1. Data present are means ± SEM, n = 3 leaves for each genotype. (D) Thermal imaging showed that cas-2 and gdsl3-1 have much lower leaf temperature compared to cas-1 and Col-0 plants. (E, F) Southern blotting confirmed that a 4770 bp region containing MPK12 and BYPASS2 between R3 and F21 was deleted in cas-2 and gdsl3-1 mutants. Genomic DNAs extracted from cas-2, gdsl3-1 and Col-0 were digested with HindIII and BamHI. The probe was set in the region F3 and F2 marked in E. (G) mpk12-4 mutant from gdsl3-1×Col-0 backcross F2 offsprings, in which MPK12-BYPASS2 was deleted but contained GDSL3, displayed similar responses to CO2 changes as gdsl3-1 by gas exchange analyses. Data present are means ± SEM, n = 3 leaves for each genotype. (H) Time-resolved relative stomatal conductance analyses showed that expression of MPK12 under the control of UBQ10 promoter in gdsl3-1 complemented the insensitive stomatal CO2 responses. Data present are means ± SEM, n = 3 leaves for each genotype. The raw data for panels (B-C), (G-H) can be found in S1 Data file. (TIF) [file pbio.2000322.s002.tif]

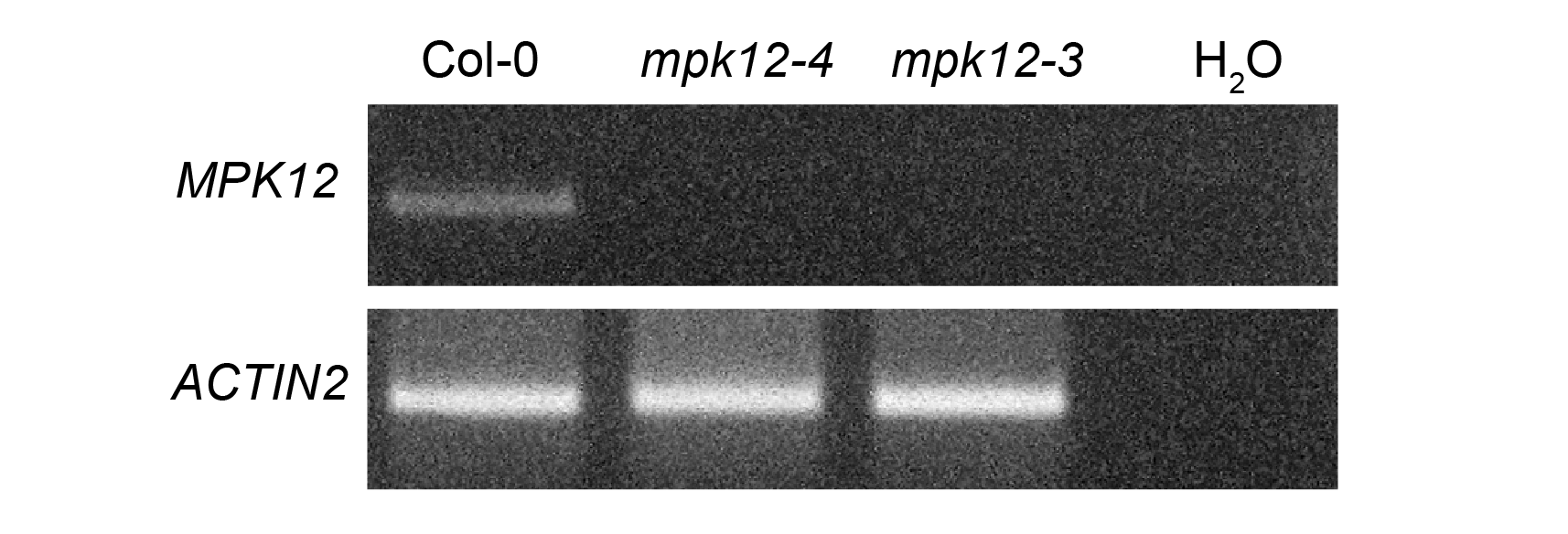

Supplement: S3 Fig — ACTIN2 was amplified as a control. (TIF) [file pbio.2000322.s003.tif]

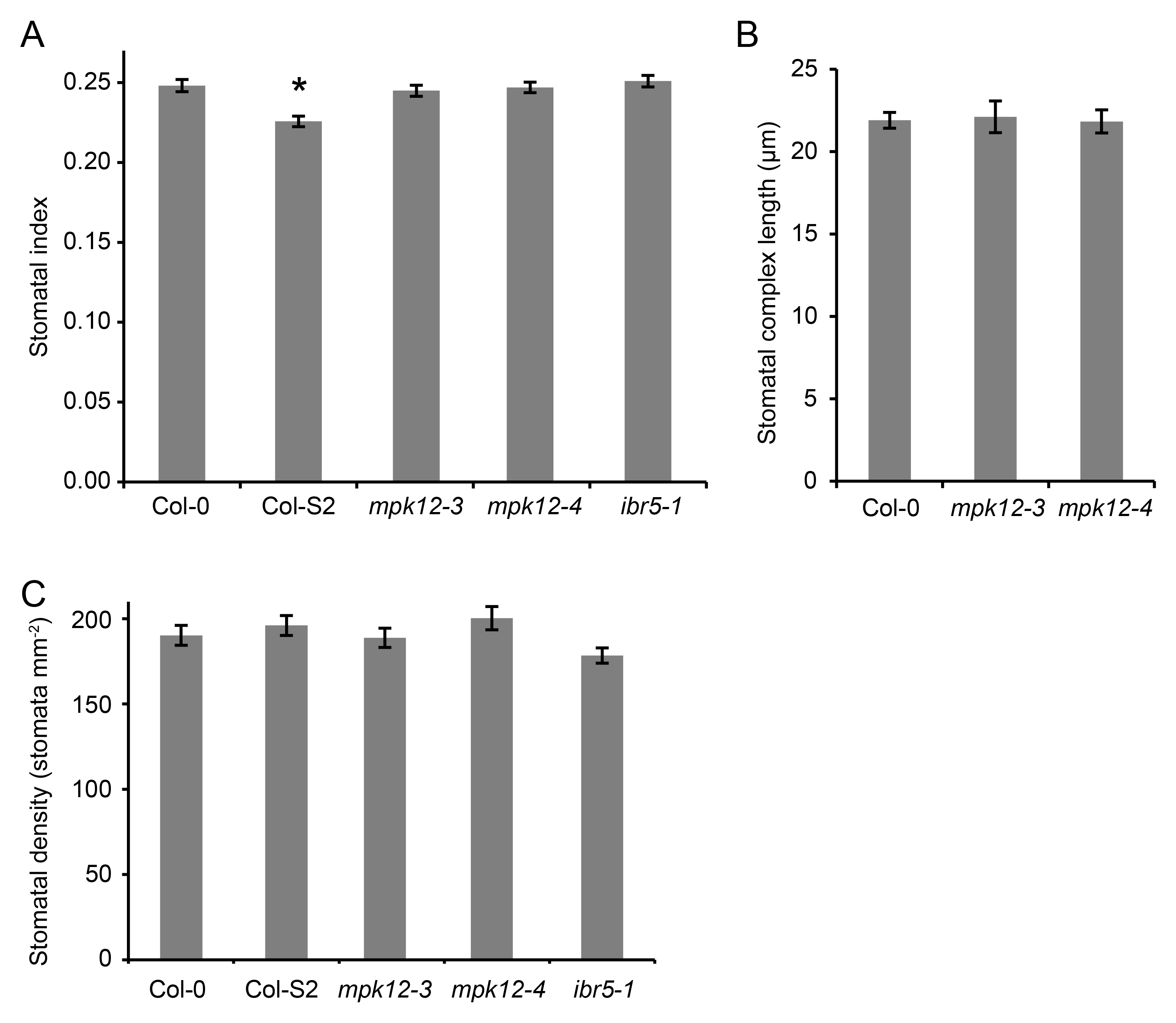

Supplement: S4 Fig — (A) Stomatal index of studied lines (mean ± SEM; 1-way ANOVA, Tukey HSD post hoc test). Experiment was repeated twice (n = 81–84 plants). (B) Stomatal complex length of mpk12 lines (mean ± SEM; 1-way ANOVA). Sample size was 4–6 plants, altogether 84–126 stomatal complexes per line were measured. (C) Stomatal density of studied lines (mean ± SEM; 1-way ANOVA). Experiment was repeated twice (n = 81–84 plants). The raw data for panels (A-C) can be found in S1 Data file. (TIF) [file pbio.2000322.s004.tif]

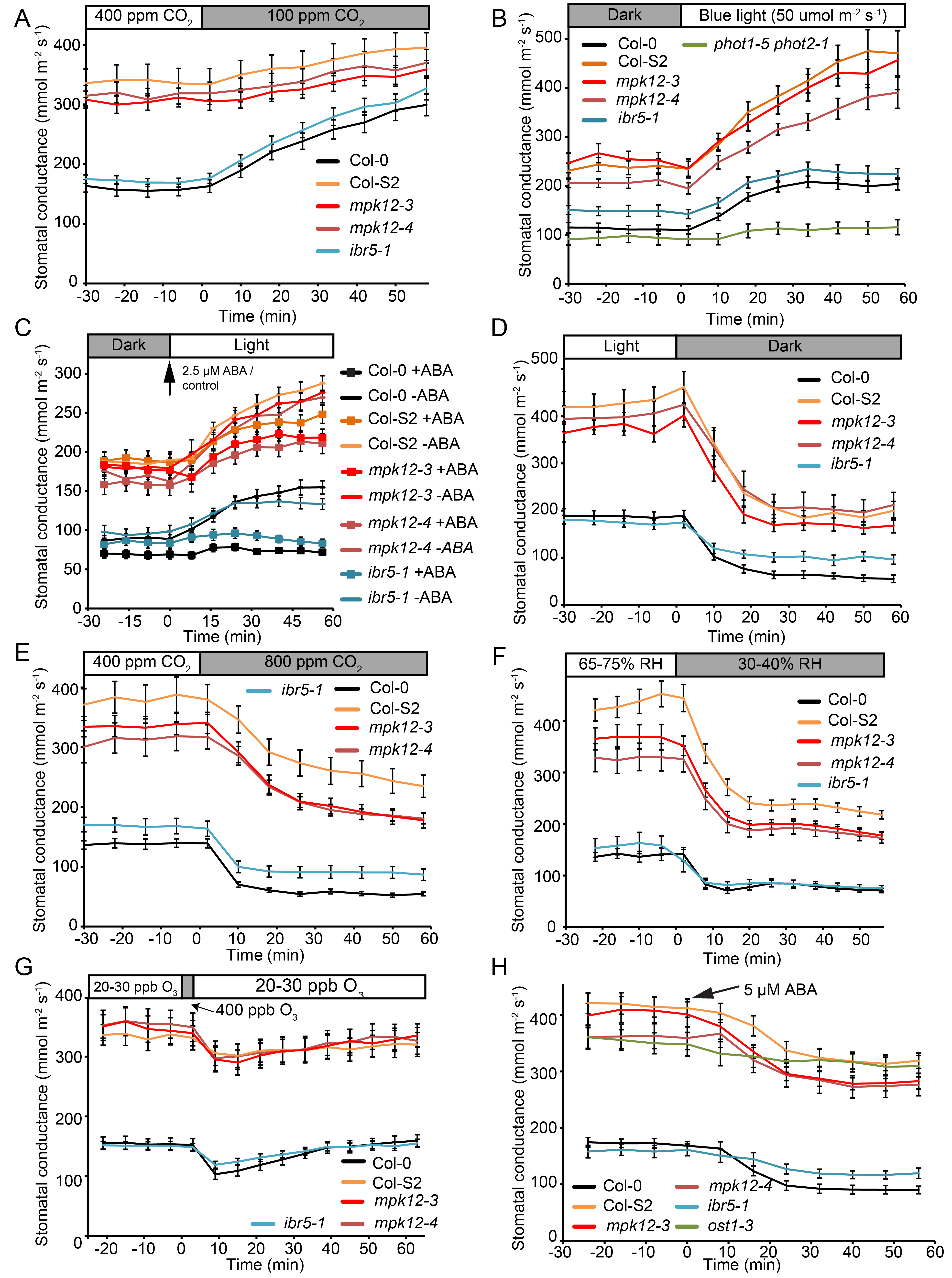

Supplement: S5 Fig — Various stimuli were applied as indicated by the bars or arrows in the legends of each panel. Stomatal opening induced by 100 ppm CO2 (A) and 50 μmol m-2s-1 blue light (B). ABA inhibited light-induced stomatal opening (C). Stomatal closure in response to darkness (D), 800 ppm CO2 (E), decrease in air humidity (F), a 3-minute O3 pulse (G) and spraying the rosette with 5 μM ABA solution (H). The data in all the figures is represented as mean ± SEM. All experiments were repeated at least three times (n = 11–18). The raw data for panels (A-H) can be found in S1 Data file. (TIF) [file pbio.2000322.s005.tif]

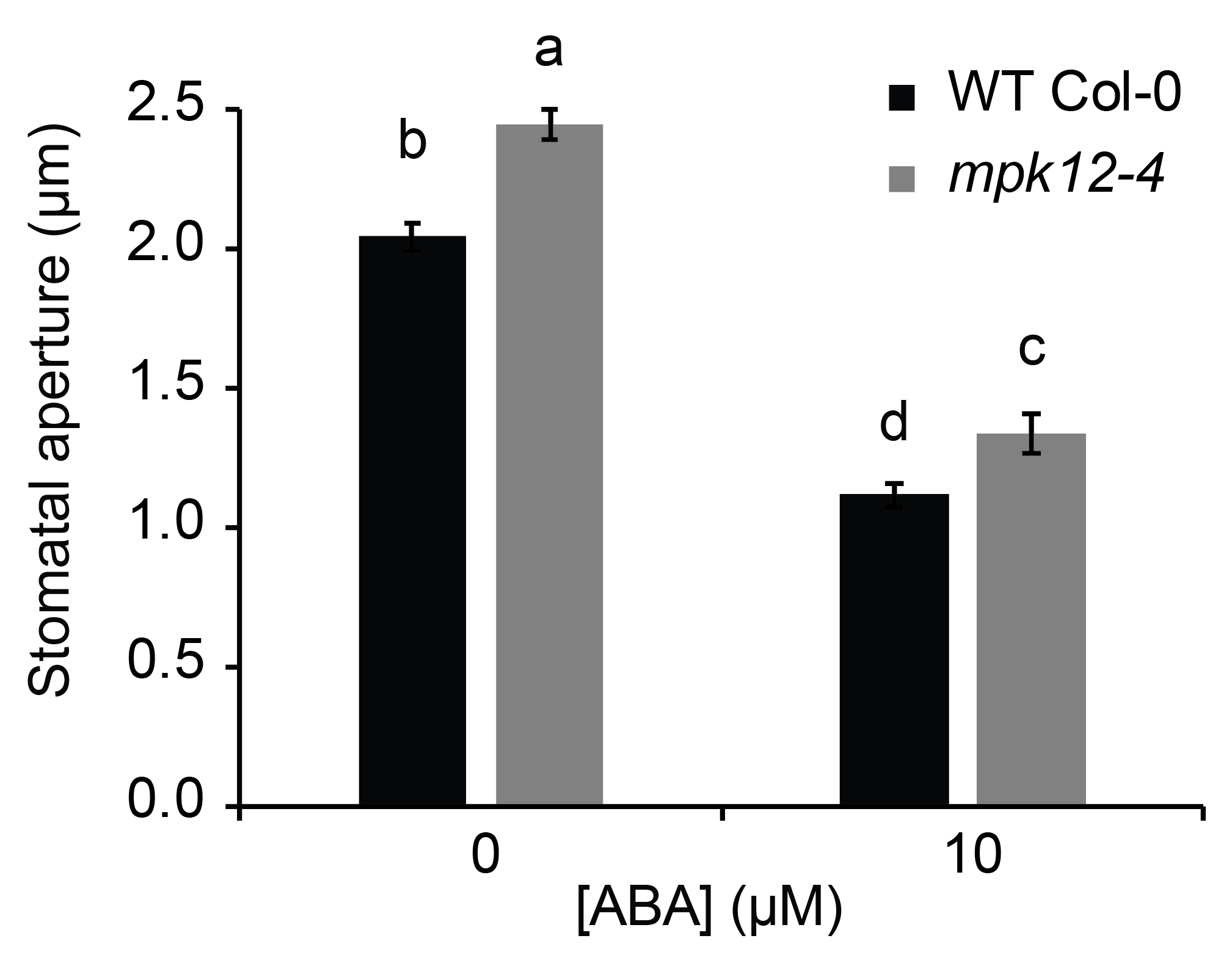

Supplement: S6 Fig — The stomata in the MPK12 deletion mutant mpk12-4 closed after treatment with 10 μM ABA for 30 min, similar as in wild type. Data are average of 3 experiments, 10 stomata per experiment and condition. Small letters denote statistically significant differences according to 2-way ANOVA with Tukey HSD post hoc test. The raw data for the figure can be found in S1 Data file. (TIF) [file pbio.2000322.s006.tif]

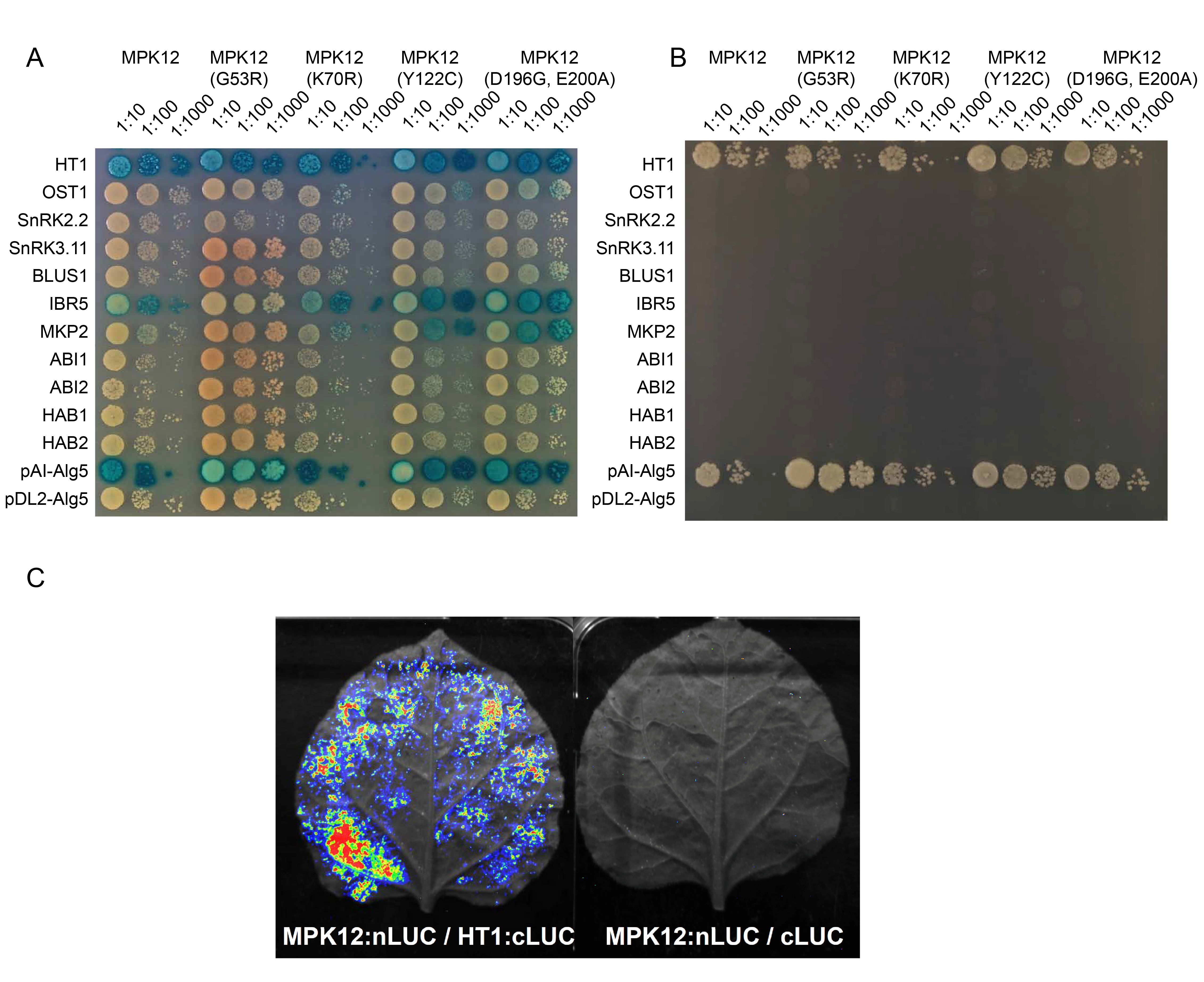

Supplement: S7 Fig — Split-ubiquitin yeast two-hybrid assays with MPK12 and different versions of MPK12 with amino acid substitutions; MPK12 G53R with the same point mutation as in Cvi-0, MPK12 K70R kinase inactive version, MPK12 Y122C and MPK12 D196G, E200A constitutively active kinase versions. (A) Yeast growth observed on SD-leu-trp plate without 3-amino-1,2,4-triazole (3-AT), 24 hours of X-Gal incubation. (B) Yeast growth observed on SD-leu-trp-his-ade plate with 20 mM 3-AT. (C) Split luciferase complementation assays showed that MPK12 interacts with HT1 in tobacco leaves. MPK12:nLUC with only cLUC was used as negative control, and showed no luciferase bioluminescence signal. (TIF) [file pbio.2000322.s007.tif]

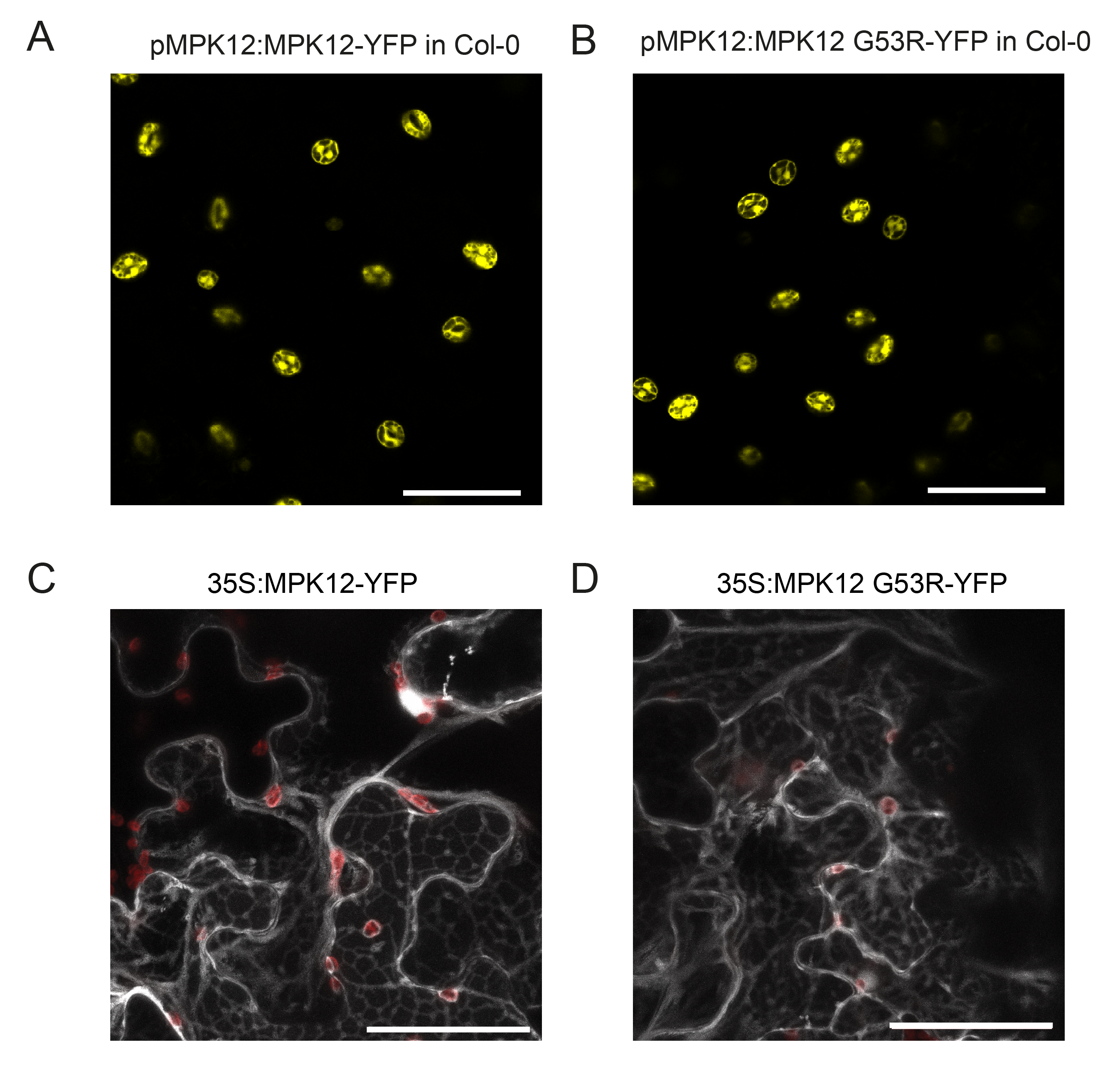

Supplement: S8 Fig — Expression of MPK12-YFP (A) and MPK12 G53R-YFP (B) under native MPK12 promoter in A. thaliana Col-0. Transient expression under the CaMV35S promoter was also shown for MPK12-YFP (C) and MPK12 G53R-YFP in N. bethamiana (D). Scale bar = 50 μm. (TIF) [file pbio.2000322.s008.tif]

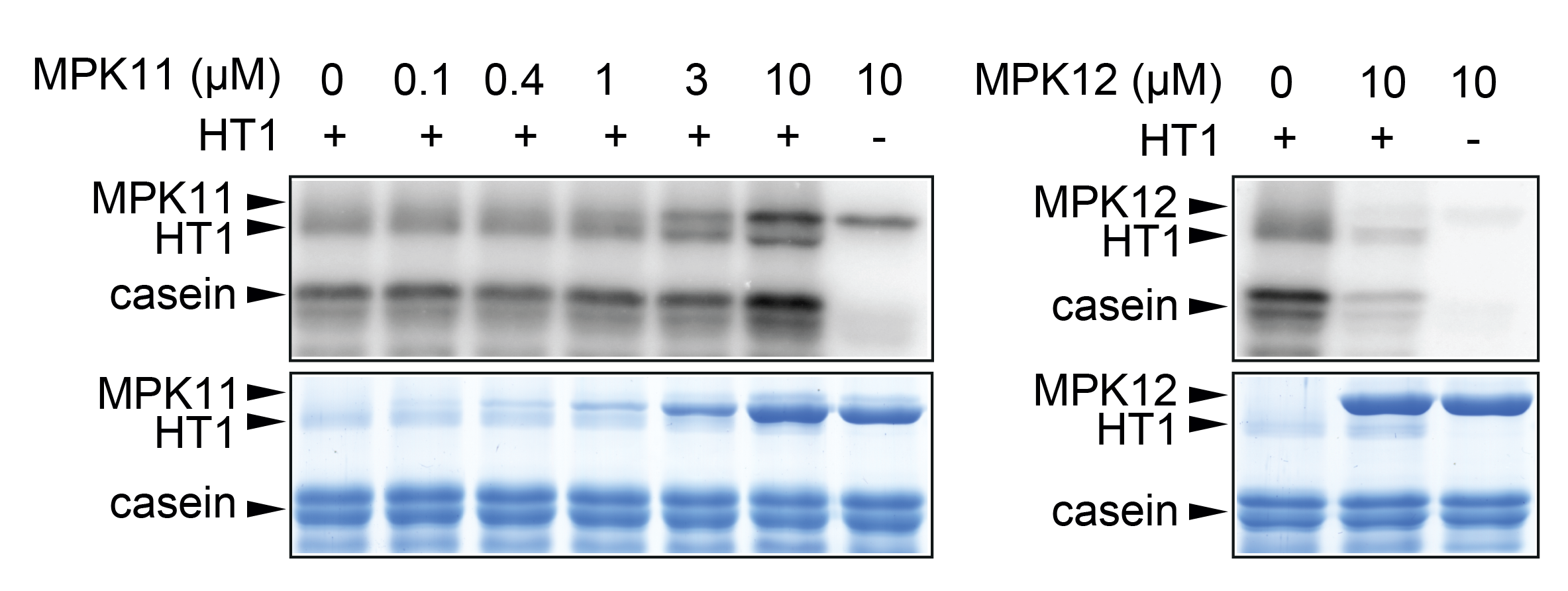

Supplement: S9 Fig — MPK11, an MPK from the same group as MPK12, was not able to inhibit HT1 showing that not all the Arabidopsis MPKs are inhibitors of HT1. This experiment was repeated four times. (TIF) [file pbio.2000322.s009.tif]
