# Supplementary material for: Natural Variation in Arabidopsis Cvi-0 Accession Reveals an Important Role of MPK12 in Guard Cell CO2 Signaling
Source: PLoS Biol. 2016 Dec 6;14(12):e2000322. doi: 10.1371/journal.pbio.2000322 (PMC5147794; doi:10.1371/journal.pbio.2000322)
Supplement: S1 Table — (DOCX) [file pbio.2000322.s010.docx]

**S1 Table.** Primers used in this study.

Chromsome 2 Cvi-0 mapping primers

AT2G42890 for GGGCTATTGAATCATTCAAACCAA HindIII CAPS marker

AT2G42890 rev GTTATGTATCCTACCCTGCTCA

AT2G44040 for CGCATATTTCCCTCTCTCAAGAC XmaIII CAPS marker

AT2G44040 rev GTCTTACGACATGGATCAGGTGC

AT2G44980 for AGAACTCTTCTTGACATGATCG TaqI CAPS Marker

AT2G44980 rev AGTGTAACGGATGGTTGGGT

AT2G45580 for CTTCGTTTTGCTGTGTGAGATCCA TaqI CAPS Marker

AT2G45580 rev ACCTGGCTCGTGCTTCCTTC

MPK12 for CTCCAGTCACTGAGTTCACCGCAG AciI CAPS marker

MPK12 rev GTTGTACCGACACACGGTGGCC

AT2G46450 for GCGTGTAAGCTAGCTCCCTTTTCG HindIII CAPS marker

AT2G46450 rev TGCAATGTTTCCTCCATGCCGC

AT2G46530 Cvi for GTCAAGTGTATACTAGACATCAGTAACT Amplifies only Cvi-0

AT2G46530 Col for GTCAAGTGTATAATATAGTTAACCAACA Amplifies only Col-0

AT2G46530 rev GGTCAATTATTGTGAGAACGAATGTG

AT2G46670 Cvi for GAGTAATAGAAGATGTAGGAGTTTGG Amplifies only Cvi-0

AT2G46670 Col for GAGTAATAGAAGACGTACGTCCTAAT Amplifies only Col-0

AT2G46670 rev CGTTGTGAAAACCTTAATTGCTAAAAC

AT2G46880 for CCTGCAATATTGTATGTGGTGTTTTAGG VspI CAPS marker

AT2G46880 rev GGTTAGATACTGATAAAATTGTAGAATATACC

Double mutant genotyping primers

ht1-2 for AGGATCCCAACACACAAGGA PsuI CAPS marker

ht1-2 rev CATCTCGTCGTTCAAAAGCA

mpk12-4 (cis) for GCACATGAAAACACGACGAGAG Primers placed on each side of the deletion

mpk12-4 (cis) rev aacaaagactacgccaatcgac Primers placed on each side of the deletion

abi1-1 for AAGATGCTGTTTCGACTATACC NcoI CAPS marker

abi1-1 rev TTTCTCCTTAGCTATCTCCTCC

MPK12 complementation constructs

MPK12_gen_GW_F: AAAAAGCAGGCTTTCAGGTGTCTAGCAAAATGA

MPK12_gen_GW_R AGAAAGCTGGGTGGGCTTACGATATGGATAAATAT

MPK12_HA_R AGAAAGCTGGGTGTGGTCAGGATTGAATTTGA

Identification of cas alleles and gsdl3-1

GABILb ATATTGACCATCATACTCATTGC

CASLP TCCTCATGAAGCTAAAGCTGC

CASRP TTGGAGCCTTTGTTGATTTTC

GDSL3LP ATTGTCATTGGTCCTCTGTGC

GDSL3RP TTTGTCCACAACCTCAAATCC

casRP4 TCAGTCGGAGCTAGGAAGGAA

casLP1 ATGGCTATGGCGGAAATGGC

LB1_SAIL GCCTTTTCAGAAATGGATAAATAGCCTTGCTTCC

Salk lba TGGTTCACGTAGTGGGCCATCG

Amplification of probe for Southern blot

F3 CCGCAATGACAGCATCTGCACC

R4 TGATCGCTTCACCTTTCGCGGT

Markers for cis rough mapping (All are SSLP markers)

Chromosome I

UPSC_1-1565 for GAATCATGAGCCGACCCTTA

UPSC_1-1565 rev GCAAACCTGCAAAACCCTTA

UPSC_1-8660 for GCGGCACAACCTAAATGAAA

UPSC_1-8660 rev TGCATGCAATTATCACGTATG

ciw12 for CTTTCAAAAGCACATCACA

ciw12 rev AGGTTTTATTGCTTTTCACA

T27K12 for GGAGGCTATACGAATCTTGACA

T27K12 rev GGACAACGTCTCAAACGGTT

nga 280 for GGCTCCATAAAAAGTGCACC

nga 280 rev CTGATCTCACGGACAATAGTGC

UPSC_1-29617 for CCCGATAATCTTCCCCAACT

UPSC_1-29617 rev GATGGCCGACGAGTACAAAT

Chromosome II

nga1145 for CCTTCACATCCAAAACCCAC

nga1145 rev GCACATACCCACAACCAGAA

nga 1126 for CGCTACGCTTTTCGGTAAAG

nga 1126 rev GCACAGTCCAAGTCACAACC

UPSC_2-18415 for AATGGGACAAAATGGGTGAA

UPSC_2-18415 rev ATTCATTGCTGTTGCGGTTT

Chromosome III

nga162 for CTCTGTCACTCTTTTCCTCTGG

nga162 rev CATGCAATTTGCATCTGAGG

UPSC_3-9633 for TTCAGCAACCTTCGATAAATCA

UPSC_3-9633 rev CCATTGCCACCGTAGAAACT

CDC2BG for ATTGAACTGTGTTGGTTTCTGG

CDC2BG rev GGGAAAAACGAAGTGACGTG

nga112 for CTCTCCACCTCCTCCAGTACC

nga112 rev TAATCACGTGTATGCAGCTGC

Chromosome IV

UPSC_4-689 for TTGTTTCCACCATCTCAATACTCT

UPSC_4-689 rev GGCCGTTGTTCTCTTCGAGT

UPSC_4-2821 for GAACTAATTTCTCGTAAAACAAACAA

UPSC_4-2821 rev AGCACATTGCTTGCATAAACT

UPSC_4-6222 for CAGAACCAAGCTGCAATGAA

UPSC_4-6222 rev CCTTCGATGTCTTCGCTGAT

ciw7 for AATTTGGAGATTAGCTGGAAT

ciw7 rev CCATGTTGATGATAAGCACAA

UPSC_4-17322 for TCCTAGATTCGTGGGGTTTG

UPSC_4-17322 rev CGCTTAAAGCTGCAAGAACC

Chromosome V

nga249 for GGATCCCTAACTGTAAAATCCC

nga249 rev TACCGTCAATTTCATCGCC

UPSC_5-7134 for AACGCCGGAGTTAGTCGAT

UPSC_5-7134 rev CGTATATGGATTGCGTGACAA

UPSC_5-17178 for AAACTGGAGTGGGACAATCG

UPSC_5-17178 rev TGACGTCGACAAATCTGTAGTAA

UPSC_5-22317 for GCATTGAAATAGTGTTTTTAACCAAA

UPSC_5-22317 rev TGTTGGTTGCCACCTTATCA

MPK12 gene expression

Act2 F GGTGATGGTGTGTCT

Act2 R ACTGAGCACAATGTTAC

MPK12 F GCGGATCCATGTCTGGAGAATCAAGCTC

MPK12 R GCGAATTCTCAGTGGTCAGGATTGAATTTG

Y2H constructs

MPK12/pDHB1 MPK12_Col, Cvi; At2g46070 Y2H bait

MPK12 Y2H for AAGAACGCGGCCATTACGGCCATGTCTGGAGAATCAAGCTCTG

MPK12 Y2H rev1 CCCCGACATGGCCGAGGCGGCCAAGTGGTCAGGATTGAATTTGACAG

MPK12(K70R) site directed mutatgenesis Y2H bait

1st PCR 1st PRODUCT: GAAAGTGGCTATTAGGAAGATCGGTAATG

1st PCR 1st PRODUCT: GCGAATTCTCAGTGGTGATGGTGATGGTGGTCAGGATTGAATTTG

1st PCR 2nd PRODUCT: GCGGATCCATGTCTGGAGAATCAAGCTC

1st PCR 2nd PRODUCT: CATTACCGATCTTCCTAATAGCCACTTTC

2nd PCR: AAGAACGCGGCCATTACGGCCATGTCTGGAGAATCAAGCTCTG

2nd PCR: CCCCGACATGGCCGAGGCGGCCAAGTGGTCAGGATTGAATTTGACAG

MPK12(Y122C) site directed mutatgenesis Y2H bait

1st PCR 1st PRODUCT: GTCTACATTGTCTGTGAGTTAATGGAC

1st PCR 1st PRODUCT: GCGAATTCTCAGTGGTGATGGTGATGGTGGTCAGGATTGAATTTG

1st PCR 2nd PRODUCT: GCGGATCCATGTCTGGAGAATCAAGCTC

1st PCR 2nd PRODUCT: GTCCATTAACTCACAGACAATGTAGAC

2nd PCR: AAGAACGCGGCCATTACGGCCATGTCTGGAGAATCAAGCTCTG

2nd PCR: CCCCGACATGGCCGAGGCGGCCAAGTGGTCAGGATTGAATTTGACAG

MPK12(D196G+E200A) site directed mutatgenesis Y2H bait

1st PCR 1st PRODUCT: CGACACAGGCTTCATGACTGCATATGTCG

1st PCR 1st PRODUCT: GCGAATTCTCAGTGGTGATGGTGATGGTGGTCAGGATTGAATTTG

1st PCR 2nd PRODUCT: GCGGATCCATGTCTGGAGAATCAAGCTC

1st PCR 2nd PRODUCT: CGACATATGCAGTCATGAAGCCTGTGTCG

2nd PCR: AAGAACGCGGCCATTACGGCCATGTCTGGAGAATCAAGCTCTG

2nd PCR: CCCCGACATGGCCGAGGCGGCCAAGTGGTCAGGATTGAATTTGACAG

HT1/pDHB1 HT1; At1g62400 Y2H prey

HT1 Y2H for CGGCCATTACGGCCATGTCTGGTTTATGTTTCAATCCG

HT1 Y2H rev1 CCCCGACATGGCCGAGGCGGCCAAGGCATTTACAGGAACAGAGGAGGA

MPK12/pPR3-N MPK12_Col, Cvi; At2g46070 Y2H bait

MPK12 Y2H for AAGAACGCGGCCATTACGGCCATGTCTGGAGAATCAAGCTCTG

MPK12 Y2H rev2 ACATGGCCGAGGCGGCCTCAGTGGTCAGGATTGAATTTG

MPK11/pPR3-N MPK11; At1g01560 Y2H bait

MPK11 Y2H for GAGTGGCCATTACGGCCCCTCAACTCACGATG

MPK11 Y2H rev GAGAGGCCGAGGCGGCCTACAACACACGCAC

HT1/pPR3-N HT1; At1g62400 Y2H prey

HT1 Y2H for CGGCCATTACGGCCATGTCTGGTTTATGTTTCAATCCG

HT1 Y2H rev2 ACATGGCCGAGGCGGCCTAGGCATTTACAGG

OST1/pPR3-N OST1; At4g33950 Y2H prey

OST1 for GAGTGGCCATTACGGCCATGGATCGACCAGCAGTG

OST1 rev GAGAGGCCGAGGCGGCCTCACATTGCGTACACAAT

SnRK2.2/pPR3-STE SnRK2.2; At3g50500 Y2H prey

SnRK2.2 for ATATGGCCATTACGGCCGTATGGATCCGGCGAC

SnRK2.2 rev TAGAGGCCGAGGCGGCCAGAGCATAAACTATCT

SnRK3.11/pPR3-STE SnRK3.11; At5g35410 Y2H prey

SnRK3.11 for ATATGGCCATTACGGCCGTATGACAAAGAAAAT

SnRK3.11 rev TAGAGGCCGAGGCGGCCACAAACGTGATTGTTC

BLUS1/pPR3-N BLUS1; At4g14480 Y2H prey

BLUS1 for ACGCGGCCATTACGGCCATGGCTCGGAACAAGC

BLUS1 rev ACATGGCCGAGGCGGCCTTAACCCAAAACACTATCTTTA

IBR5/pPR3-N IBR5; At2g04550 Y2H prey

IBR5 for ACGCGGCCATTACGGCCATGAGGAAGAGAGAAAGAGAGA

IBR5 rev ACATGGCCGAGGCGGCCCTAAGAGCCATCCATTGCAA

MKP2/pPR3-N MKP2; At3g06110 Y2H prey

MKP2 for GAGTGGCCATTACGGCCATGGAGAAAGTGGTTGATC

MKP2 rev GAGAGGCCGAGGCGGCCGTCATGCATTACCTTGGATGGA

ABI1/pPR3-STE ABI1; At4g26080 Y2H prey

ABI1 for ATATGGCCATTACGGCCTTATGGAGGAAGTATCTC

ABI rev TAGAGGCCGAGGCGGCCTTCAAGGGTTTGCTCT

ABI2/pPR3-STE ABI2; At5g57050 Y2H prey

ABI2 for ATATGGCCATTACGGCCTTATGGACGAAGTTTCTC

ABI2 rev TAGAGGCCGAGGCGGCCTTCAAGGATTTGCTCT

HAB1/pPR3-STE HAB1; At1g72770 Y2H prey

HAB1 for ATATGGCCATTACGGCCGTATGGAGGAGATGAC

HAB1 rev TAGAGGCCGAGGCGGCCACGGTTCTGGTCTTGA

HAB2/pPR3-STE HAB2; At1g17550 Y2H prey

HAB2 for ATATGGCCATTACGGCCGAATGGAAGAGATTTC

HAB2 rev TAGAGGCCGAGGCGGCCGATCTGGTCTTGAACT

BiFC constructs

35S:MPK12-YFPc/pCAMBIA1390 MPK12 variants

1st PCR for: AACTCACGATGTCTGGAGAATC

1st PCR rev: ACACACGCACGTGGTCAGGA

2nd PCR for: AATTCTCTAACTCACGATG

2nd PCR rev: CTCACCATTAACACACGCAC

35S:MPK11-YFPc/pCAMBIA1390 MPK11; At1g01560

1st PCR for: AACTCACGATGTCAATAGAGAAACCATTC

1st PCR rev: ACACACGCACAGGGTTAAACTTGACTG

2nd PCR for: AATTCTCTAACTCACGATG

2nd PCR rev: CTCACCATTAACACACGCAC

35S:HT1-YFPn/pCAMBIA1390 HT1; At1g62400

1st PCR for: AACTCACGATGTCTGGTTTATG

1st PCR rev: ACACACGCACGGCATTTACAGG

2nd PCR for: AATTCTCTAACTCACGATG

2nd PCR rev: CTCACCATTAACACACGCAC

Protein expression

HT1/pET28a

HT1 for GCCATATGATGTCTGGTTTATGTTTCAATC

HT1 rev GCGAATTCCTAGGCATTTACAGGAACAG

HT1(K113M) site directed mutagenesis:

1st PCR 1st PRODUCT for: GCCATATGATGTCTGGTTTATGTTTCAATC

1st PCR 1st PRODUCT rev: GGATCCTCACCATCATCACGGCAACGGC

1st PCR 2nd PRODUCT for: GCCGTTGCCGTGATGATGGTGAGGATCC

1st PCR 2nd PRODUCT rev: GCGAATTCCTAGGCATTTACAGGAACAG

2nd PCR for: GCCATATGATGTCTGGTTTATGTTTCAATC

2nd PCR rev: GCGAATTCCTAGGCATTTACAGGAACAG

MPK12/pET28a MPK12_Col, Cvi; At2g46070

MPK12 for GCGGATCCATGTCTGGAGAATCAAGCTC

MPK12 rev GCGAATTCTCAGTGGTCAGGATTGAATTTG

MPK12(K70R) site directed mutagenesis

1st PCR 1st PRODUCT for: GCGGATCCATGTCTGGAGAATCAAGCTC

1st PCR 1st PRODUCT rev: CATTACCGATCTTCCTAATAGCCACTTTC

1st PCR 2nd PRODUCT for: GAAAGTGGCTATTAGGAAGATCGGTAATG

1st PCR 2nd PRODUCT rev: GCGAATTCTCAGTGGTCAGGATTGAATTTG

2nd PCR for: GCGGATCCATGTCTGGAGAATCAAGCTC

2nd PCR rev: GCGAATTCTCAGTGGTCAGGATTGAATTTG

MPK12(Y122C) site directed mutatgenesis

1st PCR 1st PRODUCT for: GCGGATCCATGTCTGGAGAATCAAGCTC

1st PCR 1st PRODUCT rev: GTCCATTAACTCACAGACAATGTAGAC

1st PCR 2nd PRODUCT for: GTCTACATTGTCTGTGAGTTAATGGAC

1st PCR 2nd PRODUCT rev: GCGAATTCTCAGTGGTCAGGATTGAATTTG

2nd PCR for: GCGGATCCATGTCTGGAGAATCAAGCTC

2nd PCR rev: GCGAATTCTCAGTGGTCAGGATTGAATTTG

MPK11/pET28a MPK11; At1g01560

MPK11 for GCGGATCCATGTCAATAGAGAAACCATTC

MPK11 rev GCCTCGAGTTAAGGGTTAAACTTGACTG
